# TLR4 Cross-Talk With NLRP3 Inflammasome and Complement Signaling Pathways in Alzheimer's Disease

**DOI:** 10.3389/fimmu.2020.00724

**Published:** 2020-04-23

**Authors:** Junling Yang, Leslie Wise, Ken-ichiro Fukuchi

**Affiliations:** Department of Cancer Biology and Pharmacology, University of Illinois College of Medicine at Peoria, Peoria, IL, United States

**Keywords:** TLR4, Alzheimer's disease, inflammasome, complement, amyloid, synapse

## Abstract

Amyloid plaques, mainly composed of abnormally aggregated amyloid β-protein (Aβ) in the brain parenchyma, and neurofibrillary tangles (NFTs), consisting of hyperphosphorylated tau protein aggregates in neurons, are two pathological hallmarks of Alzheimer's disease (AD). Aβ fibrils and tau aggregates in the brain are closely associated with neuroinflammation and synapse loss, characterized by activated microglia and dystrophic neurites. Genome-wide genetic association studies revealed important roles of innate immune cells in the pathogenesis of late-onset AD by recognizing a dozen genetic risk loci that modulate innate immune activities. Furthermore, microglia, brain resident innate immune cells, have been increasingly recognized to play key, opposing roles in AD pathogenesis by either eliminating toxic Aβ aggregates and enhancing neuronal plasticity or producing proinflammatory cytokines, reactive oxygen species, and synaptotoxicity. Aggregated Aβ binds to toll-like receptor 4 (TLR4) and activates microglia, resulting in increased phagocytosis and cytokine production. Complement components are associated with amyloid plaques and NFTs. Aggregated Aβ can activate complement, leading to synapse pruning and loss by microglial phagocytosis. Systemic inflammation can activate microglial TLR4, NLRP3 inflammasome, and complement in the brain, leading to neuroinflammation, Aβ accumulation, synapse loss and neurodegeneration. The host immune response has been shown to function through complex crosstalk between the TLR, complement and inflammasome signaling pathways. Accordingly, targeting the molecular mechanisms underlying the TLR-complement-NLRP3 inflammasome signaling pathways can be a preventive and therapeutic approach for AD.

## Introduction

Alzheimer's disease (AD) is characterized by two neuropathological hallmarks, extracellular amyloid β (Aβ) deposits in the brain parenchyma (amyloid plaques) and cerebral blood vessels (cerebral amyloid angiopathy, CAA) and abnormal aggregates of hyperphosphorylated tau protein in brain neurons (neurofibrillary tangles, NFTs). Amyloid plaques and NFTs are accompanied with neuroinflammation including activated microglia and increased levels of cytokines (1). Profound loss of neurons and synapses is also found in AD dementia. Except a small subset of early-onset familial AD cases, the causes for the vast majority of AD cases are unknown and satisfactory therapeutic and preventive measures for AD are unavailable. Therefore, an urgent need exists to identify the molecular mechanisms that increase the risk for the vast majority of AD cases and to develop the preventive and therapeutic measures. Increasing lines of evidence indicate that central and systemic inflammation promotes AD progression and even initiates neurodegeneration (2–7). Indeed, recent genetic studies on late-onset AD have discovered about a dozen risk alleles that modulate innate immune activities and are highly expressed in brain-resident macrophages, microglia, highlighting the importance of immune responses and microglia in the pathogenesis of late-onset AD (8–10). Aging is the largest known risk factor for AD and represents chronic, systemic inflammation (inflamm-aging) (6, 11–13). Additionally, almost all highly ranked, modifiable risk factors for AD such as diabetes, obesity, hyperlipidemia, and hypertension are characterized by chronic, systemic inflammation (14–19). Inflammation caused by certain bacterial and viral infections is a risk factor of dementia (20–23). However, the precise molecular mechanisms by which inflammation increases the risk of AD remain to be elucidated. Here we discuss the impact of three innate immune signaling pathways including TLR4, NLRP3 inflammasome, and complement on the pathogenesis of AD.

## TLRs and its signaling pathways

**Figure 1:**
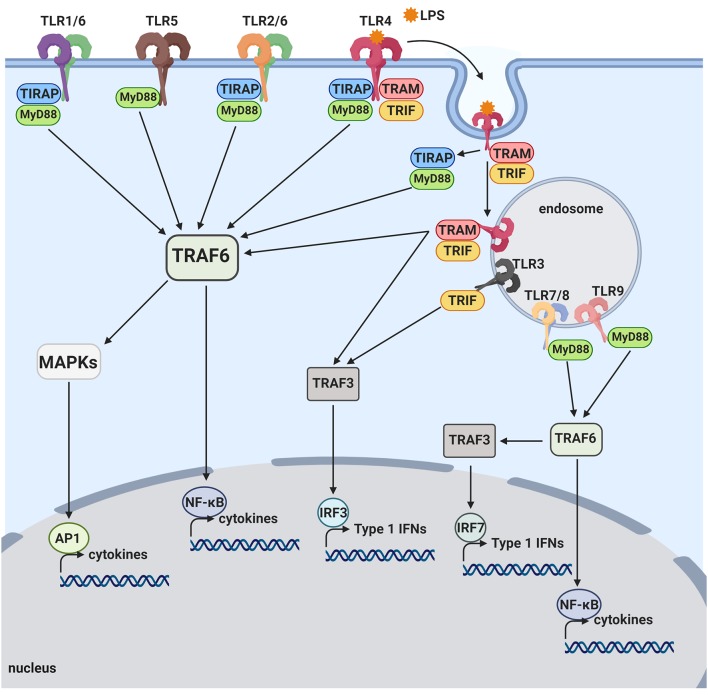
Toll-like receptor pathways. TLR1, TLR2, TLR4, TLR5, and TLR6 are mostly expressed on the cell surface and bind to bacterial products. When activated by LPS, TLR4 is internalized onto an endosome surface. The internalization triggers the release of TIRAP/MyD88, activating the TRAF6 pathway and resulting in activation of transcription factors, NF-_k_B and AP-1. The release of TIRAP/MyD88 from TLR4 allows for the signaling by TRAM/TRIF to commence from the endosome, also activating NF-_k_B as well as the transcription factor, IRF3. TLR3, TLR7, TLR8, and TLR9 are located on internal vesicles and bind to bacterial and viral nucleic acids. TLR7, TLR8, and TLR9 each activate NF-_k_B, as well as the transcription factor, IRF7, through the MyD88 pathway. TLR3 is the only toll-like receptor that does not activate via the MyD88 pathway and instead activates NF-_k_B and IRF3 through the TRIF pathway.

In responses to a variety of invading pathogens and tissue damages, the innate immune system initiates inflammatory responses through activation of pattern recognition receptors (PRRs) (24). PRRs recognize pathogen-associated molecular patterns (PAMPs), conserved structures commonly identified among different microorganisms, as well as damage-associated molecular patterns (DAMPS), molecules shed by injured cells. Currently identified classes of PRR families comprise the Toll-like receptors (TLRs) and C-type lectin receptors (CLRs), the Retinoic acid-inducible gene (RIG)-I-like receptors (RLRs) and the nucleotide-binding oligomerization domain (NOD)-Leucine Rich Repeats (LRR)-containing receptors (NLRs), and secreted proteins such as complement proteins (25, 26). TLRs are composed of an extracellular and cytoplasmic domain that belongs to a type I transmembrane receptor and recognize TLR ligands through the extracellular domain. TLR ligands can be either exogenous (PAMPs) or endogenous (DAMPs). At least 10 and 12 functional TLRs have been reported in human and mouse, respectively. The activation of TLRs by TLR ligands initiates both innate and adaptive immune responses (25, 27). TLR ligation initiates a signaling cascade that leads to activation of transcription factors that upregulate a number of target genes encoding cytokines, chemokines, growth factors, and other inflammatory mediators. Activation of TLR by pathogens and injured cells also induces phagocytic activities of macrophages/microglia and clears pathogens, damaged tissues and buildup wastes (28–31). The cytoplasmic domain of TLRs is termed Toll/interleukin-1 (IL-1) receptor (TIR) domain. TLR activation by TLR ligands initiates interaction of TLR's TIR domain with TIR domains of adaptors such as MyD88 and TRIF. Different TLRs utilize distinctive adaptor molecules, resulting in different signaling responses (Figure 1). TLR1, TLR2, TLR4, TLR5, and TLR6 are located on the cell surface membrane and recognize mostly bacterial products. TLR3, TLR7, TLR8, and TLR9 sense mostly bacterial and viral nucleic acids and are localized to intracellular vesicles including the endoplasmic reticulum, endosomes, lysosomes, and endolysosomes (32) All TLRs, with the exception of TLR3, use MyD88 as an adaptor. The ligation of TLR2 and TLR4 culminates in activation of transcription factors, NF-κB and AP1, through the MyD88-dependent pathway that is essential for expression of cytokines, chemokines and co-stimulatory molecules, such as TNF-α, IL-1β, IL-6, IL-8, IL-12, and MIP1α. TLR3 and TLR4 ligation can mediate signaling through the MyD88-indepenent (TRIF-dependent) pathway, leading to the activation of interferon regulatory factor 3 (IRF3). The activation of IRF3 induces expression of type I interferon (IFN) genes such as IFNβ and IFN-inducible genes (Figure 1). TLR3 and TLR4 ligation can activate NF-κB, also, via the TRIF-dependent pathway, resulting in induction of inflammatory cytokines (Figure 1). In TRIF-dependent signal transduction, the TLR4- lipopolysaccharide (LPS) complex on the plasma membrane is internalized to endosomes, where it triggers TRIF-dependent signal transduction (33). Importantly, although robust expression of inflammatory cytokines via MAP kinase and NF-kB activation is achieved by synergistic activation of both TRIF-dependent and MyD88-dependent pathways, TLR4 ligands can produce type I IFN solely through TRIF-dependent pathway activation (27, 34). TLR9 and TLR7 ligation can activate both IRF7 and NF-κB, leading to induction of type I IFNs and inflammatory cytokines, respectively [Figure 1; (25, 27)]. TLR signaling produce a number of genes involved in phagocytosis and inflammation through activation of transcription factors such as NF-κB, IRF3 and IRF7 (25, 35, 36).

Neurodegenerative diseases are characterized by progressive loss of specific synapses and neurons as well as abnormally aggregated proteins such as Aβ in AD (amyloid plaques) and α-synuclein in Parkinson's disease (Lewy bodies). Microglia are the principal innate immune cells in the CNS and modulate brain development, homeostasis and neuroinflammation in diseases and aging. Microglia express multiple classes of PRRs including all TLRs and respond to a variety of PAMPs and DAMPs through PRRs (37). DAMPs released from damaged or degenerating neurons and abnormally aggregated Aβ and α-synuclein (38, 39) activate microglia via PRRs, which may modulate progression of neurodegenerative diseases. Since aggregated Aβ has been shown to activate innate immune cells by interacting with several TLRs (see below), it may be possible to reduce Aβ load and neuronal injuries in the AD brain by regulating TLR signaling. However, it remains to be determined which TLR signaling pathways and effectors are involved in modulation of Aβ deposition, clearance and neuronal injuries in the brain.

## Role of TLR4 signaling in Alzheimer's disease brain

Large-scale genome-wide association studies on late-onset AD have discovered a dozen genetic risk alleles that are involved in immune/inflammatory responses and highly expressed in microglia, highlighting the importance of microglial inflammatory responses in the pathogenesis of late-onset AD. Such risk loci include APOE, TREM2, CLU, CR1, MS4A6A, MS4A4E, CD33, ABCA7, EPHA1, HLA-DRB5 & DRB1, INPP5D, and MEF2C (8, 9). Their potential roles and functions in TLR4-complement-NLRP3 signaling, are summarized in Table 1. Particularly, APOE (43), CD33 (47), INPP5D (57), and TREM2 (66) have been shown to negatively regulate TLR4 signaling. CR1 can inhibit inflammasome activation by suppressing the complement activation pathways (52). However, activation of microglial CR1 induces neurotoxic cytokines and reactive oxygen species (53). Although TREM2 is found to upregulate complement components during aging (69), it can inhibit inflammasome activation (67). CD33 may induce NLRP3 inflammasome assembly (48). APOE (46) and CLU (50) inhibit complement activation and reduce inflammation.

**Table 1:** AD risk genes involved in inflammatory responses.

**Genes**	**TLR4**	**References**	**Inflammasome**	**References**	**Complement**	**References**	**Functions**
ABCA7	No		No		No		Involved in lipid homeostasis; enhances Aβ clearance by macrophages (40, 41)
APOE	Yes	(42–44)	Maybe	(45)	Maybe	(46)	Involved in lipid metabolism (42–44)
CD33	Yes	(47)	Maybe	(48)	Maybe	(49)	Inhibitory receptor exclusive to immune cells (47)
CLU	No		No		Yes	(50)	Inhibitor of complement receptors (50)
CR1	Yes	(51)	Yes	(52)	Yes	(53)	Influences complement cascade; binds C1q; inhibits formation of MAC (52, 54)
EPHA1	No		No		No		Promotes permeability of the blood-brain barrier (55, 56)
HLA-DRB1	No		No		No		Creates beta chain 1 of the MHC class II protein complex
HLA-DRB5	No		No		No		Creates beta chain 5 of the MHC class II protein complex
INPP5D	Yes	(57, 58)	No		No		Binds DAP12 which inactivates the TREM2-DAP12 signaling complex (59)
MEF2C	Maybe	(60)	No		No		Regulates apoptosis of T cells and is necessary for transcriptional activation of IL-2 (61, 62)
MS4A cluster (MS4A4E and MS4A6A)	Maybe	(63)	No		Maybe	(63)	Ligand binding promotes calcium conductance; may modulate TREM2 expression (and TLR/complement through TREM2) (63)
TLR4 variant (rs4986790)	Yes		Yes		No		Altered ability to recruit MyD88 and TRIF (64)
TREM2	Yes	(65, 66)	Maybe	(67)	Yes	(68, 69)	Found on myeloid cells and alters inflammatory functions (70)

Previously, a coding variant of TLR4 (rs4986790) was reported to increase longevity and reduce an AD risk in Italian cohorts (71, 72). Recently, this observation has been confirmed in independent cohorts (Quebec Founder Population and presymptomatic individuals with a parental history of AD), demonstrating the association of the TLR4 variant with a reduced AD risk, better visuospatial and constructional skills, an increased cortical thickness in visual cortices, and stable IL-1β levels in cerebrospinal fluid (CSF) over time (73). Additionally, certain TLR4 gene variants are associated with an increased risk of AD in the Chinese population (74–76). These associations of TLR4 with AD in different populations indicate an important role of TLR4 in the AD pathogenesis.

Microglia, brain resident phagocytes in the innate immune system, are thought to be macrophages in the central nervous system. Fibrillar Aβ deposits are closely associated with activated microglia in the brain (1). Microglia interact with fibrillary Aβ through their cell surface receptor complexes leading to Aβ phagocytosis and inflammation. Using cultured microglia, the receptor complexes of microglia, which recognize Aβ fibrils, have been shown to contain TLR2, TLR4 and their co-receptor, CD14, as indispensable constituents of the receptor (77–79). Activation of microglia by TLR2, TLR3, TLR4, TLR7, and TLR9 ligands boosts ingestion and/or clearance of Aβ by microglia *in vitro* (78, 80–84). In line with these *in vitro* experiments, an acute (one-time) injection of LPS, a TLR4 ligand, into the brains of AD mouse models activated microglia and decreased Aβ plaques (85–87). Additionally, activation of microglia by intracerebroventricular injection of CpG-oligodeoxynucleotides (ODN), a TLR9 ligand, reduced brain Aβ deposits and ameliorated cognitive deficits in Tg2576 mice (an AD mouse model) (80, 88–91). However, sustained brain injection of LPS induced premature cerebral Aβ deposits and cognitive impairments in AD mouse models (92–94).

APP/PS1 mice (an AD mouse model) homozygous for a loss-of-function mutation (*Tlr*^*Lps*−*d*^*/Tlr*^*Lps*−*d*^) of TLR4 had greater cerebral Aβ load and poorer spatial learning than APP/PS1 mice with TLR4 wild-type alleles (81, 95). AD mouse models show increases in brain cytokine levels including TNF-α, IL-1β, IL-17, and IL-10. Such increases in the brain cytokines were abolished in APP/PS1 mice with the TLR4 mutation, indicating TLR4-dependent upregulation of the cytokines in APP/PS1 mice (96). However, TLR4-dependent upregulation of cytokines and microglial activation were not observed in young APP/PS1 mice before Aβ deposition (95, 96). Additionally, TLR2 deficiency in an AD mouse model [APPSwe/PS1(A246E)] increased brain Aβ42 levels (toxic form of Aβ) and accelerated spatial and contextual memory impairments (97). These *in vivo* data suggest that activation of certain TLRs can be therapeutic option for AD. However, APP/PS1 mice defective for CD14 (CD14 gene knockout), a co-receptor for TLR4, showed a decrease in Aβ plaques (98). MyD88 deficiency decreased cerebral Aβ load and improved behavioral deficits in APP/PS1 mice (99). Additionally, transplantation of bone marrow cells with MyD88 deficiency in an AD mouse model ameliorated brain Aβ levels and cognitive deficits much better than MyD88-sufficient bone marrow cells (100). The latter experiments indicate that activation of certain TLRs can be detrimental to the AD progression. These experimental results also indicate that the *in vitro* data can be misleading perhaps due to oversimplification of the *in vitro* systems as well as difficulties in mimicking chronic activation of TLRs in the *in vitro* systems. Accordingly, *in vivo* experiments in detail in TLR ligand treatment regimen, age, sex and genetic background of experimental animals are indispensable for a better understanding of the roles of the TLR signaling pathways in the AD pathogenesis.

## Role of TLR4 signaling in systemic inflammation in Alzheimer's disease (AD)

There are increasing lines of evidence that systemic inflammation promotes AD progression and initiates microglial activation and neurodegeneration (2–7). Aging is the largest known risk factor for AD and is characterized by chronic, systemic low-grade inflammation, referred to as “inflamm-aging” (11–13). Additionally, highly ranked, modifiable risk factors for AD such as depression, hypertension, diabetes, obesity, and hyperlipidemia are characterized by a chronic, systemic low-grade inflammation (14–19). For example, visceral adipose tissue of obese subjects contains innate and adaptive immune cells and shows low-grade chronic inflammation, which is identified as a major contributor to the advancement of metabolic diseases including type 2 diabetes mellitus and coronary heart diseases (101, 102). Indeed, when a diabetic AD mouse model was produced by crossing APP23 mice (an AD model) with leptin-deficient (ob/ob) mice, the onset of diabetes exacerbated cognitive deficits, cerebral amyloid angiopathy, and cerebrovascular inflammation (103). A high-fat diet increased insoluble cerebral Aβ and soluble tau in the brains of 3xTg-AD mice (an AD model) (104). Low-grade inflammation plays a pivotal role in the initiation, progression, and propagation of the atherosclerotic process (105, 106). Atherogenic diet exacerbated cognitive deficits and cerebral Aβ deposits in Tg2576 mice (an AD mouse model) and the aortic atherosclerotic lesion area positively correlated with cerebral Aβ deposits (107). Certain peripheral, as well as CSF inflammatory markers, such as IL-6 and C-reactive protein (CRP) have been reported to forecast dementia or decline in cognitive functions many years before their onset (106, 108–113). These AD risk factors have been shown to be associated with altered TLR4 signaling. The TLR4 +896A/G coding variant (rs4986790) is underrepresented in patients with myocardial infarction, Alzheimer's disease or prostate cancer, whereas it is more frequently found in centenarians in Italian and Canadian cohorts (71–73). Their blood samples produce less IL-6, TNF-α, and eicosanoids (PGE2 and LTB4) in response to LPS, compared to other TLR4 genotypes (114). Anti-aging effects of caloric restriction is associated with downregulation of the TLR4/MyD88/NF-κB pathway in rodents (115). Apolipoprotein E (ApoE)-deficient mice are prone to high-fat diet-induced atherosclerosis, which is reduced in additional TLR4-deficiency or MyD88-deficiency, indicating an important role of TLR4/MyD88 signaling in atherosclerosis (116). Activation of TLR4 contributes to insulin resistance by impairing insulin signal transduction via inhibitory phosphorylation on serine residues in insulin receptor substrate (IRS) (117). Therefore, these AD risk factors may contribute to the AD development via TLR4 signaling.

Systemic infections are also associated with AD although not all studies found such associations. Infection of certain bacteria including *Helicobacter pylori, Porphyromonas gingivalis, Chlamydia pneumonia*, and *Borrelia burgdorferi*, has been found to be risk factors for the development of dementia (20–22, 118, 119) In an AD mouse model (APP/PS1 mice), *Bordetella pertussis* respiratory challenge led to T cell infiltration into the brain and increased microglial activation and Aβ deposition (120). Peripheral injections of TLR ligands such as LPS and poly I:C, TLR4 and TLR3 ligand, into animals and humans have been commonly implemented to mimic bacterial and viral infections, respectively. Repeated peripheral LPS injection in wild type mice led to cognitive deficits and increases in cerebral Aβ levels and apoptotic cells (121, 122). A single intravenous poly I:C injection into 4-month-old 3xTg-AD mice increased cerebral Aβ deposits and altered tau phosphorylation at age 15 months. Additionally, systemic exposure to poly I:C during late gestation in wild type mice increased cerebral APP (Aβ precursor protein) levels, altered tau phosphorylation and cognitive function in old ages and these phenotypic alterations were exacerbated when the prenatal exposure was followed by a second challenge during their adulthood (123). Repeated systemic injection of LPS induced premature cerebral Aβ deposits and cognitive impairments in AD mouse models (92–94). Repeated intraperitoneal injection of LPS activated microglia and increased tau phosphorylation in an AD mouse model (3xTg-AD) (124). Daily intraperitoneal LPS injection in Kunming mice for 7 days induced microglia activation, upregulation of proinflammatory cytokines (both mRNA and protein) including IL-1β, TNF-α, and IL-6, synapse loss, and impairment of learning and memory (125). Acute intraperitoneal LPS injection also increased tau phosphorylation in the hippocampal neurons of C57BL/6 mice (126, 127). Furthermore, periodontitis evoked by inoculation of *Porphyromonas gingivalis* exacerbated brain Aβ deposition and cognitive deficits in an AD mouse model (J20 PDGF-APPSw-Ind mice) (128). Repeated intraperitoneal injection of LPS derived from *Porphyromonas gingivalis* induced cognitive deficits, intraneuronal Aβ accumulation, microglial activation, and increases in IL-1β in middle-aged (12 months) wild-type C57BL/6 mice but not in young (2 months) mice (129). These findings support the hypothesis that systemic inflammation promotes AD progression and even initiates AD-like pathological changes. Indeed, peripheral LPS administration has been widely used to model neuroinflammation and neurodegenerative diseases including AD in rodents and the lists of such experimental models are found in the following review papers (130–133). Importantly, TLR4 in brain-resident immune cells plays a predominant role in sustained neuroinflammation including IL-1β upregulation, which is induced by systemic LPS administration rather than TLR4 in peripheral immune cells (134). However, the precise mechanisms by which systemic inflammation contributes to AD initiation and progression remain to be elucidated.

So far, as we discussed above, almost all chronic, systemic inflammatory events predominantly exert pro-inflammatory responses in brain microglia, leading to exacerbation of neurodegenerative diseases including AD. Recently, Wendeln et al. (135) reported that one-time peritoneal injection of LPS prior to brain Aβ deposition (at 3 months of age) in an AD mouse model primed microglia and exacerbated the brain Aβ load 6 months later while 4 consecutive peritoneal injections of LPS (0.5 mg/kg) induced tolerance and reduced the Aβ load. Additionally, chronic intraperitoneal administration of CpG-ODN (TLR9 ligand) and monophosphoryl lipid A (MPL, TLR4 ligand) reduced Aβ plaques and NFTs, and restored cognitive deficits in AD mouse models (80, 88–91). However, the precise mechanisms, by which the repeated TLR ligand treatments improve AD-like pathophysiology are unclear. One possible explanation is that the repeated TLR ligand treatments increase stress resistance or adaptation/tolerance of microglia, leading to reduced inflammatory responses of microglia, alleviation of AD-like pathology, and cognitive deficits (136). It is important to understand that systemic inflammatory events as well as peripheral treatment with TLR ligands can shape the phenotype of microglia in the CNS. These results suggest that modulation of brain microglial phenotype by peripheral treatment with certain TLR ligands at appropriate doses and treatment intervals can be therapeutic and/or preventive to AD.

## NLRP3 inflammasome and aging

**Figure 2:**
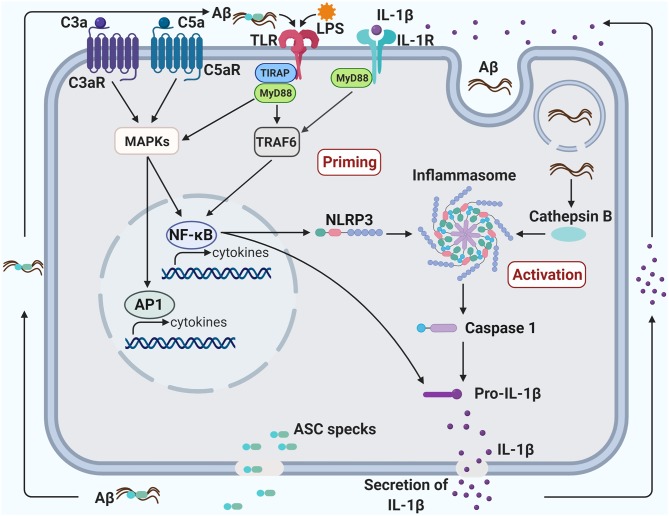
Crosstalk between TLR4, NLRP3 inflammasome, and complement promotes neuroinflammation in Alzheimer's disease. Priming of the inflammasome occurs when the transcription factor, NF-_k_B, is activated, triggering the production of both NLRP3 and Pro-IL-1β. NF-_k_B can be activated via the TLR/IL-1R MyD88-dependent pathway and the C3/C5 MAPK pathway. The TLR pathway can be induced by a bacterial component, such as LPS, and the MAPK pathway can be induced by C3a/C5a binding to their respective receptors. The activation of NF-_k_B through complement, TLR and IL-1R pathways may create a synergistic increase in pro-inflammatory factors. The inflammasome can be activated in several ways, including an increase of endogenous damage-associated and pathogen-associated molecular patterns or an efflux of potassium or chloride ions. Additionally, aggregated fibrillary Aβ engulfed by the microglia can damage the lysosome and leak into the cytoplasm, also contributing to the activation of the inflammasome. Activation of the inflammasome can induce pyroptosis, leading to the secretion of IL-1β and ASC specks. ASC specks bind to Aβ and seed the surrounding parenchyma leading to further Aβ aggregation. Aggregated Aβ can also bind to TLR and induce activation of the MyD88 pathway. Likewise, IL-1β secreted from the pyroptotic microglia can bind to IL-1R and induce activation of the MyD88 pathway. The induction of the MyD88 pathway through the by-products of microglial pyroptosis may lead to a vicious cycle of inflammasome priming, inflammasome activation and pyropotosis that will exacerbate Aβ pathology.

Inflammasomes consist of multimeric protein complexes in the cytoplasm, which mediate activation of IL-1β and IL-18 and induce pyroptosis, a programmed cell death. Inflammasomes are involved in initiation and sustainment of the innate immune response (137). The NLRP3 inflammasome consists of a sensor (NLRP3), and adaptor (ASC or PYARD) and an effector (caspase 1) (138). Activation of the NLRP3 inflammasome and the production of IL-1β are tightly regulated and require two triggering steps, a priming step and an activation step (Figure 2). In the priming step, expression of the inflammasome components (NLRP3, caspase 1 and pro-IL-1β) needs to be upregulated to their suitable expression levels for inflammasome activation. This upregulation can be induced by various PAMPs or DAMPs, including LPS or amyloid, respectively, through activation of PRRs and cytokine receptors, including TLRs and IL-1R, respectively (138). In the activation step, NLRP3 can be activated by a large number of stimuli such as endogenous DAMPs, PAMPs, efflux of potassium (K^+^) or chloride (Cl^−^) ions and flux of calcium ions (Ca^2+^) (138).

The biggest risk factor for Alzheimer's disease is advanced age (139). Aging is characterized by systemic low-grade inflammation, referred to as “inflamm-aging” (11–13) and senescent cells are characterized by the senescence-associated secretory phenotype (SASP), indicating proinflammatory characteristics including increased secretion of IL-1β, IL-6, IL-8, TGF-β, and TNF-α (140). IL-1β production increases during aging in the mouse brain, which is exacerbated by intraperitoneal injection of LPS (1 mg/kg), (141, 142). IL-1β, IL-6, TGF-β, and TNF-α levels are elevated in AD brain tissue, as well as in AD patients' CSF and serum (143). Fibrillar Aβ induces more IL-1β production in microglia isolated from aged mice than those derived from young mice (144). NLRP3 deficiency ameliorates central and peripheral low-grade inflammation and SASP and improves cognitive function and motor performance in aged mice (141). IL-1R deficiency (Il1r^−/−^) also ameliorates cognitive decline associated with aging in mice (141). Thus, inhibition of NLRP3 inflammasome can be a therapeutic and preventive target for age-related chronic diseases including AD.

## Role of NLRP3 inflammasomes in Alzheimer's disease brain

Fibrillary Aβ can induce IL-1β release from cultured microglia in an NLRP3-dependent and ASC-dependent manner, where NLRP3 serves as a sensor of aggregated Aβ for inflammasome activation (145). ASC deficiency decreases brain Aβ deposits and improves cognitive deficits in APP/PS1 mice. Injection of ASC specks induces spreading of Aβ deposits in APP/PS1 mice. However, this is not observed in ASC-deficient APP/PS1 mice, and co-administration of anti-ASC antibody blocks the spreading of Aβ pathology. Thus, ASC specks released from pyroptotic microglia induce seeding and spreading of Aβ oligomers and aggregates, leading to AD progression (146). NLRP3 or caspase-1 deficiency in APP/PS1 mice leads to reduced brain caspase-1 and IL-1β activation, increased microglial Aβ phagocytosis, reduced brain Aβ load, and protection of neuronal spine loss, long-term potentiation (LTP) decline, and cognitive deficits (147). However, the reduced Aβ load in NLRP3-deficient APP/PS1 mice is discernible at 16 months of age but not at 4 months of age (147). In patients with early AD or mild cognitive impairment due to AD, levels of IL-1β and caspase-1 activity are significantly increased (147, 148) and ASC-bound Aβ is found in AD patients' brains (146). These observations suggest that NLRP3 inflammasome activation represents an early pathogenic event in AD. Intrastriatal injection of fibrillar Aβ in mice causes microglial activation, which is inhibited in mice with MyD88 deficiency, ASC deficiency, caspase-1 deficiency, or IL-1R deficiency (145), suggesting that aggregated Aβ initiates a signaling cascade involving MyD88, NLRP3 inflammasome, and IL-1β. In line with these observations, MyD88-deficiency decreases microglial activation and cerebral Aβ load and improves behavioral deficits in APP/PS1 mice (99, 149). Moreover, MyD88 deficiency enhances Aβ phagocytosis by microglia/macrophages *in vitro* and bone marrow reconstitution by MyD88-deficient cells reduces Aβ load and improves cognitive functions more efficiently compared with MyD88-sufficient cells in AD mouse models including APP/PS1 and TgCRND8 mice (100). Expression levels of IL-1β mRNA and protein are upregulated in the brains of APP/PS1 mice compared to those in age-matched APP/PS1 mice with a loss-of-function TLR4 mutation at 9–15 months of age but not at 5 months (95, 96). These findings suggest that TLR4/MyD88 signaling is involved in the priming step of NLRP3 inflammasome activation in AD mouse models (Figure 2).

In addition to a crucial role of the NLRP3 inflammasome in Aβ pathophysiology in AD, tau pathology is influenced by NLRP3 activation (150). NLRP3 or ASC deficiency decreases tau hyperphosphorylation and aggregation by regulating tau kinases (GSK-3β and CaMKII-α) and phosphatases (PP2A) in Tau22 mice that express tau mutations found in frontotemporal dementia. Intracerebral injection of fibrillar Aβ-containing brain homogenates enhances tau phosphorylation and aggregation in Tau22 mice, which is blocked by NLRP3 or ASC deficiency (150), suggesting that Aβ-induced NLRP3 inflammasome activation exacerbates tau pathology in AD and its animal models.

## Role of NLRP3 inflammasomes in systemic inflammation in Alzheimer's disease

LPS is a potent TLR4 ligand and its systemic administration is widely used to model systemic inflammation. A list of animal models summarizing the effects of LPS treatment on NLRP3 inflammasome activation is found in an excellent review article by Heneka et al. (151). Several papers have reported microglial NLRP3 inflammasome activation after peripheral LPS injection. Single intraperitoneal injection of LPS (5 mg/kg) in C57BL/6 (B6) mice induced microglial activation, upregulation of NLRP3, ASC, caspase-1p10, and IL-1β in the hippocampus, leading to behavioral alterations (depression like behavior and memory deficits) for 29 days after LPS injection, which were inhibited by a NLRP3 inhibitor (152). Intraperitoneal injection of LPS (3.5 mg/kg) in B6 mice induced activation of microglia and NLRP3 inflammasome, and increased IL-1β expression in CNS, which were exacerbated by microglia-specific A20 (NF-κB inhibitor) deficiency but not by deficiency in other cell types (neuron, astrocyte, and oligodendrocytes) (153). Intraperitoneal injection of LPS (0.5 mg/kg) in B6 mice induced activation of microglia, increases in NLRP3, ASC, caspase-1 and IL-1β in the hippocampus, and depressive behavior (154) and such effects by LPS (1 mg/kg) were inhibited in NLRP3-deficient mice (155). Intraperitoneal injection of LPS (1 mg/kg) in APP/PS1 mice at 15 months of age induced decreases in Aβ uptake by microglia, increases in the number and size of Aβ deposits and in peripheral myeloid cells that infiltrated into the brain but not at 5 months of age (156). Such changes by intraperitoneal LPS injection were blocked by NLRP3 deficiency. These results suggest that systemic LPS administration induces microglial NLRP3 inflammasome activation, increased brain Aβ load and brain infiltration of peripheral myeloid cells in an age dependent manner, leading to exacerbation of AD pathophysiology.

## TLR/IL-1R/MyD88 signaling in sustained vicious circle of NLRP3 inflammasome activation in Alzheimer's disease

LPS is often used to prime NLRP3 inflammasome (157). LPS can induce canonical and non-canonical NLRP3 inflammasome activation (138). In canonical inflammasome priming, activated TLR4 by LPS signals through the adaptor protein, MyD88, culminating in activation of transcription factor, nuclear-factor-kB (NF-κB), that elevates pro-IL-1β and NLRP3 expression (158, 159). Toll-like receptors (TLRs) including TLR2, TLR4, TLR6, and their co-receptor, CD14, are indispensable constituents of the receptor complexes for microglial activation by Aβ, leading to cytokine and chemokine production (78, 79, 95). Extracellular fibrillary Aβ can prime the canonical inflammasome pathway by activating the TLR/MyD88/NF-κB signaling pathway [Figure 2; (160, 161)]. In the activation step, phagocytosed Aβ in microglia leads to lysosomal damage and liberation of cathepsin B and/or production of mitochondrial reactive oxygen species, which trigger formation of the NLRP3 inflammasome complex, causing caspase 1 activation, IL-1β production and pyroptosis (145, 162). Oligomeric and fibrillar Aβ can directly interact with NLRP3 and ASC, resulting in NLRP3 inflammasome activation, also (163). ASC specks released by microglial pyroptosis quickly bind to extracellular Aβ and induce seeding and spreading of Aβ oligomers and aggregates (146). Aggregated Aβ further promotes microglial inflammasome priming via TLR/MyD88 signaling. Additionally, secreted IL-1β also induces microglial inflammasome priming via IL-1R/MyD88 signaling (164). Thus, this vicious circle of NLRP3 inflammasome activation by TLR/IL-1R/MyD88 signaling may lead to chronic/sustained inflammation and neurodegeneration in AD (Figure 2).

## Complement in aging brain

Complements belong to the pattern recognition receptors in the innate immune system and involved in recognition and clearance of pathogens, damaged tissues, aggregated proteins, and toxic wastes (165, 166). Additionally, complement proteins have been implicated in diverse processes during brain development, aging and neurological diseases (26). Virtually all complement components are locally expressed in the brain and microglia express almost all classical complement components and their receptors including C1qR, CR3, C3aR, and C5aR (167, 168). Particularly, complement and microglia play an important role in synaptic pruning, that is, complement-tagged synapse elimination by microglia, during neural development, aging, and neurodegenerative diseases (169). In the normally developing brain, opsonization of synapses by complement factors (tagged by C1q, C3b, and C4) triggers microglial phagocytosis, resulting in elimination of the tagged synapses.

During normal brain aging in human and mouse, C1q protein levels dramatically increase in certain regions of the brain, including the hippocampus, substantia nigra, and piriform cortex. Aged mice with C1q deficiency exhibit significantly less cognitive and memory decline compared with wild-type mice (170). Marked increases in C1q levels are found in dendritic spines at synapses in the aged rhesus macaque dorsolateral prefrontal cortex as well as glia ensheathed synapses, suggesting C1q-tagged synapse elimination by glial phagocytosis as a possible mechanism for age-related degeneration (171). C57BL/6J (B6) mice (at 16 months of age) show age-dependent neuron loss in hippocampal CA3 but not in CA1, which is not observed complement C3-deficient B6 mice. Additionally, aged C3-deficient B6 mice show better cognition and LTP than wild-type B6 mice, implying that C3 is also involved in age-dependent synapse loss and cognitive decline (172).

## Role of complement in Alzheimer's disease brain

In AD, the degree of region-specific synapse loss better correlates with cognitive decline than amyloid plaques, NFTs and neuron loss (173, 174) and genetic variants of complement receptor 1 (CR1) and clusterin (CLU, apolipoprotein J), which are parts of the complement system, are identified as AD risk factors by genome wide association studies (175). Certain components of complements including C1q, C3, C4, and C5b-C9 (membrane attack complex, MAC) accumulate in amyloid plaques and NFT in the brains of AD patients (176–179). A positive correlation is found between expression levels of C3 and C3a receptor (C3aR1) in the brain and cognitive decline and Braak staging in AD patients (180). Additionally, CD57 that prevents MAC assembly is decreased in AD brain (181). CSF and plasma levels of certain complement proteins have been reported as promising biomarkers for AD diagnosis and progression (182–186). These observations suggest that activation of the complement system may contribute to the AD pathogenesis.

C1q deficiency decreases plaque-associated glial activation and mitigates progressive decreases in synaptic markers in Tg2576 mice without changes in brain Aβ load (187). In J20 mice (an AD mouse model), upregulation and deposition of C1q onto synapses precedes synaptic loss in the hippocampus before overt amyloid plaque formation (188). The toxic effects of Aβ oligomers on synapse loss and LTP inhibition are blocked by C1q deficiency or its inhibitor in mice (188). C1q tags tau-affected synapses and microglia eliminate C1q-tagged synapses by engulfment in PS19 mice (a frontotemporal dementia model). This process is inhibited by C1q-blocking antibodies (189). LPS and Aβ increases production of C3 in primary microglial cultures in a dose dependent manner (190). Aβ oligomer-induced synaptic engulfment by microglia is inhibited by CR3 deficiency in adult mice and inhibition of C3 or microglial CR3 decreases Aβ oligomer-induced synapse loss (188). C3 deficiency ameliorates age-dependent loss of synapses and neurons, and cognitive deficits in aged APP/PS1 mice although it increases cerebral Aβ deposits (191). C3 deficiency mitigates amyloid plaque-associated synapse loss in another AD model mice, PS2APP, and rescues neuron loss and LTP deficits in PS19 mice (192). Similarly, C3aR1 deficiency mitigates tau pathology, neuroinflammation, synaptic deficits and neurodegeneration in PS19 mice (180). Activation of microglia by LPS or Aβ increases sialidase activity and desialylation of the microglial surface, leading to stimulation of CR3-mediated phagocytosis of neurons by microglia in primary glial-neuronal co-cultures. This neuronal loss by microglial phagocytosis is inhibited by a blocking antibody against CD11b (a component of CR3) and a sialidase inhibitor (193). Oral administration of a C5a receptor antagonist (PMX205) decreases Aβ deposition and glial activation in Tg2576 and 3xTg mice, improves cognitive deficits in Tg2576 mice and reduces tau hyperphosphorylation in 3xTg mice (194). These observations support the hypotheses that complement activation exacerbates the AD progression and that the complement signaling pathway that regulates pruning of excess synapses by microglia during brain development is inadequately initiated and mediates synapse loss and neurodegeneration in AD.

In contrast with these hypotheses, the other investigators found beneficial effects of complement activation. C1q has been reported to have a protective effect against neurotoxic Aβ fibrils and oligomers by activating cAMP-response element-binding protein and AP-1, resulting in upregulation of LRP1B and G protein-coupled receptor 6(GPR6), in cultured neurons as well as 3xTg mice (195). Additionally, genetic deficiency of C3 increases Aβ deposition and induces neurodegeneration and alternative activation (M2) of microglia in aged J20 mice (17 months) (196). Inhibition of C3 by overexpressing soluble complement receptor related protein y (sCrry) increases Aβ deposition and neurodegeneration in J20 mice (197). These findings support the notion that activation of these complement components is neuroprotective.

## Role of complement in systemic inflammation in neurodegeneration

Intraperitoneal administration of LPS (10 mg/kg) for 7 days induces marked upregulation of C1q and C3 by activating the classical complement pathway, microglial activation, synapse loss in the hippocampus, and cognitive deficits in Kunming mice (125). Repeated intraperitoneal administration of LPS (1 mg/kg/day for 4 consecutive days) induces dopaminergic neuron loss in the substantia nigra in mice but a single LPS injection does not. This loss of dopaminergic neurons is prevented in C3-deficient mice and associated with increased expression of genes involved in the classical and alternative complement (Itgam of CR3, C4, C3, and HF1) and phagosome (Fcer2b, Fcgr3, Fcgr4, Tyrobp, and Fcer1 g) pathways in the brain, suggesting that repeated peripheral LPS administration induces complement-mediated elimination of dopaminergic neurons by microglial phagocytosis (198). Intraperitoneal injection of LPS (5 mg/kg) activates microglia and activated microglia induce A1 astrocytes by releasing TNFα, IL-1α, and C1q in B6 mice. A1 astrocytes can drive neurodegeneration by releasing a neurotoxin and multiple complement components including C1q and C3, leading to microglial CR3-mediated synapse pruning and loss (199). A1 astrocytes are abundantly observed in diverse neurodegenerative diseases including AD (199). These findings indicate that systemic inflammation can activate brain complement and microglia, leading to loss of synapses and neurons, cognitive deficits, and neurodegeneration.

## Potential complement and TLR crosstalk in neuroinflammation and Alzheimer's disease

As parts of the host defense innate immune system, TLRs and complements engage in synergistic or antagonistic signaling crosstalk to orchestrate immune responses. Indeed, most pathogens activate both TLRs and complements. TLR4 activation upregulates expression of complement components, potentially leading to complement activation (200, 201). In responses to TLR ligands including LPS (TLR4), zymosan (TLR2/6), and CpG-ODN (TLR9), mice deficient in a major membrane complement inhibitor, decay-accelerating factor (DAF), show striking elevation of plasma IL-1β, IL-6, and TNF-α in a complement-dependent manner. This synergistic effect of complement on the cytokine production by TLRs in peripheral tissues has been attributed to activation of NF-κB and mitogen-activated protein kinases (ERK1/2 and c-Jun N-terminal kinase) through the C5a-C5aR1 and C3a-C3aR signal pathways in mice [Figure 2; (200)]. Indeed, co-stimulation of human monocytes (THP-1 cell line) with aggregated Aβ and C5a markedly enhances secretion of IL-1β and IL-6 through NF-κB activation *in vitro* (202). Therefore, it is possible that activation of C5aR and C3aR signaling by C5a and C3a, respectively, synergistically enhances proinflammatory responses initiated by aggregated Aβ-induced TLR4 activation in the brain, leading to AD initiation and progression. Additionally, the formation of the complement membrane attack complex (MAC) triggers increased cytosolic Ca^2+^ concentration, resulting in mitochondrial dysfunction and NLRP3 activation that causes caspase 1 activation and IL-1β secretion *in vitro* (203), which may further promote a pathogenic cycle of the TLR4-complement-NLRP3 inflammasome interactions in AD.

In human monocytes, C5aR activation by C5a enhances LPS/TLR4-induced expression of IL-6 and TNF-α production while, in macrophages, C5a increases IL-10 secretion and inhibits LPS/TLR4-induced upregulation of IL-6 and TNF-α via C5aR/MEK/ERK signaling (204). This distinct regulation of LPS/TLR4 signaling by C5a in different cell types supports the concept that monocytes in circulation act as danger sensor and heighten inflammatory responses to PAMPs and DAMPs, while tissue macrophages restrain excess inflammation for host protection/tissue repair (204). Therefore, it is also possible that, in homeostatic/resting microglia, C5a and/or C3a synergistically enhance pro-inflammatory responses triggered by Aβ-TLR4 activation for removal of toxic Aβ aggregates while, in activated microglia, C5a and/or C3a antagonizes Aβ-TLR4-induced pro-inflammatory responses for neuroprotection. This host defense function of complement appears to be altered to host-offensive actions during aging (205). This detrimental alteration of complement-TLR signaling during aging may be exacerbated in AD.

## Concluding remarks

TLRs function as a host defense mechanism against pathogens and tissue damages. In peripheral tissues, complement and NLRP3 inflammasome modulate immune and inflammatory responses initiated by TLRs through crosstalk between their signaling pathways. TLR4 primes NLRP3 inflammasome in the peripheral tissues as well as in the central nervous system (CNS). As Aβ forms aggregates, a vicious cycle of Aβ-TLR4-NLRP3 inflammasome-IL-1β in microglia sustains neuroinflammation in AD. Systemic inflammation can exacerbate neuroinflammation and neurodegeneration in AD via TLR4 and complement activation. In the peripheral tissues, the crosstalk between TLR and complement is complex and contextual depending on cell type, tissue, species and disease models and complement seems to function as a molecular switch of TLR signaling (pro- or anti-inflammatory) and as a coordinator between innate and adaptive immune responses. However, such regulatory functions of complement have not been investigated in the CNS or brain-resident immune cells including microglia. One of the obstacles that hamper the investigation is that available microglial cell lines and primary microglia derived from the brain have characteristics different from brain resident microglia because microglia are sensitive to environmental changes. Such obstacles may be circumvented by use of new technologies such as the RiboTag and BacTRAP (Translating Ribosome Affinity Purification) methods (206, 207), single-nuclei or single cell RNAseq, genome editing tools, and iPSC-derived 3D co-culture brain models (208). Repeated failures of Aβ-targeted therapeutics indicate the need for a new approach for AD therapy and prevention based on disease mechanisms alternative to the amyloid cascade hypothesis. Inflammation and immune cells play a central role in the initiation and progression of AD. It is crucial to elucidate the molecular mechanisms by which inflammatory responses and immune cells drive the AD initiation and progression.

## Author Contributions

All authors are involved in writing and editing this manuscript and in intellectual contributions.

## Conflict of Interest

The authors declare that the research was conducted in the absence of any commercial or financial relationships that could be construed as a potential conflict of interest.

## References

[1] Akiyama H Barger S Barnum S Bradt B Bauer J. Inflammation and alzheimer's disease. Neurobiol Aging. (2000) 21:383–421. 10.1016/S0197-4580(00)00124-X 10858586 PMC3887148

[2] Holmes C. Review: systemic inflammation and alzheimer's disease. Neuropathol Appl Neurobiol. (2013) 39:51–68. 10.1111/j.1365-2990.2012.01307.x 23046210

[3] Perry VH Holmes C. Microglial priming in neurodegenerative disease. Nat Rev Neurol. (2014) 10:217–24. 10.1038/nrneurol.2014.38 24638131

[4] Misiak B Leszek J Kiejna A. Metabolic syndrome, mild cognitive impairment and alzheimer's disease–the emerging role of systemic low-grade inflammation and adiposity. Brain Res Bull. (2012) 89:144–9. 10.1016/j.brainresbull.2012.08.003 22921944

[5] Takeda S Sato N Morishita R. Systemic inflammation, blood-brain barrier vulnerability and cognitive/non-cognitive symptoms in alzheimer disease: relevance to pathogenesis and therapy. Front Aging Neurosci. (2014) 6:171. 10.3389/fnagi.2014.00171 25120476 PMC4114193

[6] Giunta B Fernandez F Nikolic WV Obregon D Rrapo E. Inflammaging as a prodrome to alzheimer's disease. J Neuroinflammation. (2008) 5:51. 10.1186/1742-2094-5-51 19014446 PMC2615427

[7] Fulop T Itzhaki RF Balin BJ Miklossy J Barron AE. Role of microbes in the development of alzheimer's disease: state of the art - an international symposium presented at the 2017 IAGG congress in san francisco. Front Genet. (2018) 9:362. 10.3389/fgene.2018.00362 30250480 PMC6139345

[8] Lambert JC Ibrahim-Verbaas CA Harold D Naj AC Sims R. Meta-analysis of 74,046 individuals identifies 11 new susceptibility loci for alzheimer's disease. Nat Genet. (2013) 45:1452–8. 10.1038/ng.2802 24162737 PMC3896259

[9] Manolio TA Collins FS Cox NJ Goldstein DB Hindorff LA. Finding the missing heritability of complex diseases. Nature. (2009) 461:747–53. 10.1038/nature08494 19812666 PMC2831613

[10] Sims R van der Lee SJ Naj AC Bellenguez C Badarinarayan N. Rare coding variants in PLCG2, ABI3, and TREM2 implicate microglial-mediated innate immunity in alzheimer's disease. Nat Genet. (2017) 49:1373–84. 10.1038/ng.3916 28714976 PMC5669039

[11] Hebert LE Weuve J Scherr PA Evans DA. Alzheimer disease in the united states (2010-2050) estimated using the 2010 census. Neurology. (2013) 80:1778–83. 10.1212/WNL.0b013e31828726f5 23390181 PMC3719424

[12] Franceschi C Campisi J. Chronic inflammation (inflammaging) and its potential contribution to age-associated diseases. J Gerontol A Biol Sci Med Sci. (2014) 69(Suppl. 1), S4–9. 10.1093/gerona/glu057 24833586

[13] Fulop T Witkowski JM Olivieri F Larbi A. The integration of inflammaging in age-related diseases. Semin Immunol. (2018) 40:17–35. 10.1016/j.smim.2018.09.003 30287177

[14] Deckers K van Boxtel MP Schiepers OJ de Vugt M Munoz Sanchez JL. Target risk factors for dementia prevention: a systematic review and delphi consensus study on the evidence from observational studies. Int J Geriatr Psychiatry. (2015) 30:234–46. 10.1002/gps.4245 25504093

[15] Wium-Andersen MK Orsted DD Nielsen SF Nordestgaard BG. Elevated C-reactive protein levels, psychological distress, and depression in 73, 131 individuals. JAMA Psychiatry. (2013) 70:176–84. 10.1001/2013.jamapsychiatry.102 23266538

[16] Hodes GE Pfau ML Leboeuf M Golden SA Christoffel DJ. Individual differences in the peripheral immune system promote resilience versus susceptibility to social stress. Proc Natl Acad Sci USA. (2014) 111:16136–41. 10.1073/pnas.1415191111 25331895 PMC4234602

[17] Canli T. Reconceptualizing major depressive disorder as an infectious disease. Biol Mood Anxiety Disord. (2014) 4:10. 10.1186/2045-5380-4-10 25364500 PMC4215336

[18] Kennedy BK Berger SL Brunet A Campisi J Cuervo AM. Geroscience: linking aging to chronic disease. Cell. (2014) 159:709–13. 10.1016/j.cell.2014.10.039 25417146 PMC4852871

[19] Salvioli S Monti D Lanzarini C Conte M Pirazzini C. Immune system, cell senescence, aging and longevity–inflamm-aging reappraised. Curr Pharm Des. (2013) 19:1675–9. 10.2174/1381612811319090015 23589904

[20] Stein PS Desrosiers M Donegan SJ Yepes JF Kryscio RJ. Tooth loss, dementia and neuropathology in the nun study. J Am Dent Assoc. (2007) 138:quiz 1381–2. 10.14219/jada.archive.2007.0046 17908844

[21] Kamer AR Craig RG Pirraglia E Dasanayake AP Norman RG. TNF-alpha and antibodies to periodontal bacteria discriminate between alzheimer's disease patients and normal subjects. J Neuroimmunol. (2009) 216:92–7. 10.1016/j.jneuroim.2009.08.013 19767111 PMC2783848

[22] Kountouras J Tsolaki M Gavalas E Boziki M Zavos C. Relationship between helicobacter pylori infection and alzheimer disease. Neurology. (2006) 66:938–40. 10.1212/01.wnl.0000203644.68059.5f 16567719

[23] Fulop T Witkowski JM Bourgade K Khalil A Zerif E. Can an infection hypothesis explain the beta amyloid hypothesis of alzheimer's disease? Front Aging Neurosci. (2018) 10:224. 10.3389/fnagi.2018.00224 30087609 PMC6066504

[24] Kawasaki T Kawai T. Toll-like receptor signaling pathways. Front Immunol. (2014) 5:461. 10.3389/fimmu.2014.00461 25309543 PMC4174766

[25] Takeuchi O Akira S. Pattern recognition receptors and inflammation. Cell. (2010) 140:805–20. 10.1016/j.cell.2010.01.022 20303872

[26] Lee JD Coulthard LG Woodruff TM. Complement dysregulation in the central nervous system during development and disease. Semin Immunol. (2019) 45:101340. 10.1016/j.smim.2019.101340 31708347

[27] Kawai T Akira S. Toll-like receptors and their crosstalk with other innate receptors in infection and immunity. Immunity. (2011) 34:637–50. 10.1016/j.immuni.2011.05.006 21616434

[28] Pineau I Lacroix S. Endogenous signals initiating inflammation in the injured nervous system. Glia. (2009) 57:351–61. 10.1002/glia.20763 18803306

[29] Kluwe J Mencin A Schwabe RF. Toll-like receptors, wound healing, and carcinogenesis. J Mol Med. (2009) 87:125–38. 10.1007/s00109-008-0426-z 19089397 PMC2791674

[30] Rakoff-Nahoum S Medzhitov R. Toll-like receptors and cancer. Nat Rev Cancer. (2009) 9:57–63. 10.1038/nrc2541 19052556

[31] Frangogiannis NG. The immune system and cardiac repair. Pharmacol Res. (2008) 58:88–111. 10.1016/j.phrs.2008.06.007 18620057 PMC2642482

[32] Blasius AL Beutler B. Intracellular toll-like receptors. Immunity. (2010) 32:305–15. 10.1016/j.immuni.2010.03.012 20346772

[33] Kagan JC Su T Horng T Chow A Akira S Medzhitov R. TRAM couples endocytosis of toll-like receptor 4 to the induction of interferon-beta. Nat Immunol. (2008) 9:361–8. 10.1038/ni1569 18297073 PMC4112825

[34] Yamamoto M Sato S Hemmi H Hoshino K Kaisho T. Role of adaptor TRIF in the MyD88-independent toll-like receptor signaling pathway. Science. (2003) 301:640–3. 10.1126/science.1087262 12855817

[35] Doyle SE O'Connell RM Miranda GA Vaidya SA Chow EK. Toll-like receptors induce a phagocytic gene program through p38. J Exp Med. (2004) 199:81–90. 10.1084/jem.20031237 14699082 PMC1887723

[36] McKimmie CS Roy D Forster T Fazakerley JK. Innate immune response gene expression profiles of N9 microglia are pathogen-type specific. J Neuroimmunol. (2006) 175:128–41. 10.1016/j.jneuroim.2006.03.012 16697053

[37] Kigerl KA de Rivero Vaccari JP Dietrich WD Popovich PG Keane RW. Pattern recognition receptors and central nervous system repair. Exp Neurol. (2014) 258:5–16. 10.1016/j.expneurol.2014.01.001 25017883 PMC4974939

[38] Kim C Ho DH Suk JE You S Michael S. Neuron-released oligomeric alpha-synuclein is an endogenous agonist of TLR2 for paracrine activation of microglia. Nat Commun. (2013) 4:1562. 10.1038/ncomms2534 23463005 PMC4089961

[39] Daniele SG Beraud D Davenport C Cheng K Yin H Maguire-Zeiss KA. Activation of MyD88-dependent TLR1/2 signaling by misfolded alpha-synuclein, a protein linked to neurodegenerative disorders. Sci Signal. (2015) 8:ra45. 10.1126/scisignal.2005965 25969543 PMC4601639

[40] Abe-Dohmae S Ikeda Y Matsuo M Hayashi M Okuhira K. Human ABCA7 supports apolipoprotein-mediated release of cellular cholesterol and phospholipid to generate high density lipoprotein. J Biol Chem. (2004) 279:604–11. 10.1074/jbc.M309888200 14570867

[41] Jehle AW Gardai SJ Li S Linsel-Nitschke P Morimoto K. ATP-binding cassette transporter A7 enhances phagocytosis of apoptotic cells and associated ERK signaling in macrophages. J Cell Biol. (2006) 174:547–56. 10.1083/jcb.200601030 16908670 PMC2064260

[42] Ali K Middleton M Pure E Rader DJ. Apolipoprotein E suppresses the type I inflammatory response *in vivo*. Circ Res. (2005) 97:922–7. 10.1161/01.RES.0000187467.67684.43 16179587

[43] Zhu Y Kodvawala A Hui DY. Apolipoprotein E inhibits toll-like receptor (TLR)-3- and TLR-4-mediated macrophage activation through distinct mechanisms. Biochem J. (2010) 428:47–54. 10.1042/BJ20100016 20218969 PMC3050041

[44] Zhang H Wu LM Wu J. Cross-talk between apolipoprotein E and cytokines. Mediators Inflamm. (2011) 2011:949072. 10.1155/2011/949072 21772670 PMC3136159

[45] Theendakara V Patent A Peters Libeu CA Philpot B Flores S. Neuroprotective sirtuin ratio reversed by ApoE4. Proc Natl Acad Sci USA. (2013) 110:18303–8. 10.1073/pnas.1314145110 24145446 PMC3831497

[46] Yin C Ackermann S Ma Z Mohanta SK Zhang C ApoE attenuates unresolvable inflammation by complex formation with activated C1q. Nat Med. (2019) 25:496–506. 10.1038/s41591-018-0336-8 30692699 PMC6420126

[47] Ishida A Akita K Mori Y Tanida S Toda M. Negative regulation of toll-like receptor-4 signaling through the binding of glycosylphosphatidylinositol-anchored glycoprotein, CD14, with the sialic acid-binding lectin, CD33. J Biol Chem. (2014) 289:25341–50. 10.1074/jbc.M113.523480 25059667 PMC4155695

[48] Sallman DA Cluzeau T Basiorka AA List A. Unraveling the pathogenesis of MDS: the NLRP3 inflammasome and pyroptosis drive the MDS phenotype. Front Oncol. (2016) 6:151. 10.3389/fonc.2016.00151 27379212 PMC4909736

[49] Son M Diamond B Volpe BT Aranow CB Mackay MC Santiago-Schwarz F. Evidence for C1q-mediated crosslinking of CD33/LAIR-1 inhibitory immunoreceptors and biological control of CD33/LAIR-1 expression. Sci Rep. (2017) 7:270. 10.1038/s41598-017-00290-w 28325905 PMC5412647

[50] Trougakos IP Gonos ES. Clusterin/apolipoprotein J in human aging and cancer. Int J Biochem Cell Biol. (2002) 34:1430–48. 10.1016/S1357-2725(02)00041-9 12200037

[51] Macsik-Valent B Nagy K Fazekas L Erdei A. Complement receptor type 1 (CR1, CD35), the inhibitor of BCR-mediated human B cell activation, differentially regulates TLR7, and TLR9 induced responses. Front Immunol. (2019) 10:1493. 10.3389/fimmu.2019.01493 31312202 PMC6614493

[52] Triantafilou M Hughes TR Morgan BP Triantafilou K. Complementing the inflammasome. Immunology. (2016) 147:152–64. 10.1111/imm.12556 26572245 PMC4717242

[53] Zhu XC Yu JT Jiang T Wang P Cao L Tan L. CR1 in alzheimer's disease. Mol Neurobiol. (2015) 51:753–65. 10.1007/s12035-014-8723-8 24794147

[54] Klickstein LB Barbashov SF Liu T Jack RM Nicholson-Weller A. Complement receptor type 1 (CR1, CD35) is a receptor for C1q. Immunity. (1997) 7:345–55. 10.1016/S1074-7613(00)80356-8 9324355

[55] Ivanov AI Romanovsky AA. Putative dual role of ephrin-eph receptor interactions in inflammation. IUBMB Life. (2006) 58:389–94. 10.1080/15216540600756004 16801213

[56] Chen F Liu Z Peng W Gao Z Ouyang H. Activation of EphA4 induced by EphrinA1 exacerbates disruption of the blood-brain barrier following cerebral ischemia-reperfusion via the rho/ROCK signaling pathway. Exp Ther Med. (2018) 16:2651–8. 10.3892/etm.2018.6460 30186497 PMC6122430

[57] An H Xu H Zhang M Zhou J Feng T. Src homology 2 domain-containing inositol-5-phosphatase 1 (SHIP1) negatively regulates TLR4-mediated LPS response primarily through a phosphatase activity- and PI-3K-independent mechanism. Blood. (2005) 105:4685–92. 10.1182/blood-2005-01-0191 15701712

[58] Keck S Freudenberg M Huber M. Activation of murine macrophages via TLR2 and TLR4 is negatively regulated by a lyn/PI3K module and promoted by SHIP1. J Immunol. (2010) 184:5809–18. 10.4049/jimmunol.0901423 20385881

[59] Peng Q Malhotra S Torchia JA Kerr WG Coggeshall KM Humphrey MB. TREM2- and DAP12-dependent activation of PI3K requires DAP10 and is inhibited by SHIP1. Sci Signal. (2010) 3:ra38. 10.1126/scisignal.2000500 20484116 PMC2900152

[60] Kim SO Ono K Tobias PS Han J. Orphan nuclear receptor Nur77 is involved in caspase-independent macrophage cell death. J Exp Med. (2003) 197:1441–52. 10.1084/jem.20021842 12782711 PMC2193909

[61] Youn HD Sun L Prywes R Liu JO. Apoptosis of T cells mediated by Ca^2+^-induced release of the transcription factor MEF2. Science. (1999) 286:790–3. 10.1126/science.286.5440.790 10531067

[62] Pan F Ye Z Cheng L Liu JO. Myocyte enhancer factor 2 mediates calcium-dependent transcription of the interleukin-2 gene in T lymphocytes: a calcium signaling module that is distinct from but collaborates with the nuclear factor of activated T cells (NFAT). J Biol Chem. (2004) 279:14477–80. 10.1074/jbc.C300487200 14722108

[63] Deming Y Filipello F Cignarella F Cantoni C Hsu S. The MS4A gene cluster is a key modulator of soluble TREM2 and alzheimer's disease risk. Sci Transl Med. (2019) 11:eaau2291. 10.1126/scitranslmed.aau2291 31413141 PMC6697053

[64] Figueroa L Xiong Y Song C Piao W Vogel SN Medvedev AE. The Asp299Gly polymorphism alters TLR4 signaling by interfering with recruitment of MyD88 and TRIF. J Immunol. (2012) 188:4506–15. 10.4049/jimmunol.1200202 22474023 PMC3531971

[65] Hamerman JA Jarjoura JR Humphrey MB Nakamura MC Seaman WE Lanier LL. Cutting edge: inhibition of TLR and FcR responses in macrophages by triggering receptor expressed on myeloid cells (TREM)-2 and DAP12. J Immunol. (2006) 177:2051–5. 10.4049/jimmunol.177.4.2051 16887962

[66] Ito H Hamerman JA. TREM-2, triggering receptor expressed on myeloid cell-2, negatively regulates TLR responses in dendritic cells. Eur J Immunol. (2012) 42:176–85. 10.1002/eji.201141679 21956652 PMC3444819

[67] Qu W Wang Y Wu Y Liu Y Chen K. Triggering receptors expressed on myeloid cells 2 promotes corneal resistance against *Pseudomonas aeruginosa* by inhibiting caspase-1-dependent pyroptosis. Front Immunol. (2018) 9:1121. 10.3389/fimmu.2018.01121 29887864 PMC5980993

[68] Sharif O Gawish R Warszawska JM Martins R Lakovits K. The triggering receptor expressed on myeloid cells 2 inhibits complement component 1q effector mechanisms and exerts detrimental effects during pneumococcal pneumonia. PLoS Pathog. (2014) 10:e1004167. 10.1371/journal.ppat.1004167 24945405 PMC4055749

[69] Linnartz-Gerlach B Bodea LG Klaus C Ginolhac A Halder R. TREM2 triggers microglial density and age-related neuronal loss. Glia. (2019) 67:539–50. 10.1002/glia.23563 30548312 PMC6590266

[70] Jay TR von Saucken VE Landreth GE. TREM2 in neurodegenerative diseases. Mol Neurodegener. (2017) 12:56. 10.1186/s13024-017-0197-5 28768545 PMC5541421

[71] Balistreri CR Colonna-Romano G Lio D Candore G Caruso C. TLR4 polymorphisms and ageing: implications for the pathophysiology of age-related diseases. J Clin Immunol. (2009) 29:406–15. 10.1007/s10875-009-9297-5 19459036

[72] Minoretti P Gazzaruso C Vito CD Emanuele E Bianchi M. Effect of the functional toll-like receptor 4 Asp299Gly polymorphism on susceptibility to late-onset alzheimer's disease. Neurosci Lett. (2006) 391:147–9. 10.1016/j.neulet.2005.08.047 16157451

[73] Miron J Picard C Lafaille-Magnan ME Savard M Labonte A. Association of TLR4 with alzheimer's disease risk and presymptomatic biomarkers of inflammation. Alzheimers Dement. (2019) 15:951–60. 10.1016/j.jalz.2019.03.012 31175027

[74] Wang LZ Yu JT Miao D Wu ZC Zong Y. Genetic association of TLR4/11367 polymorphism with late-onset alzheimer's disease in a han chinese population. Brain Res. (2011) 1381:202–7. 10.1016/j.brainres.2011.01.007 21236243

[75] Yu JT Miao D Cui WZ Ou JR Tian Y. Common variants in toll-like receptor 4 confer susceptibility to alzheimer's disease in a han chinese population. Curr Alzheimer Res. (2012) 9:458–66. 10.2174/156720512800492495 22272615

[76] Chen YC Yip PK Huang YL Sun Y Wen LL. Sequence variants of toll like receptor 4 and late-onset alzheimer's disease. PLoS ONE. (2012) 7:e50771. 10.1371/journal.pone.0050771 23272070 PMC3525588

[77] Liu Y Walter S Stagi M Cherny D Letiembre M. LPS receptor (CD14): a receptor for phagocytosis of alzheimer's amyloid peptide. Brain. (2005) 128:1778–89. 10.1093/brain/awh531 15857927

[78] Reed-Geaghan EG Savage JC Hise AG Landreth GE. CD14 and toll-like receptors 2 and 4 are required for fibrillar A{beta}-stimulated microglial activation. J Neurosci. (2009) 29:11982–92. 10.1523/JNEUROSCI.3158-09.2009 19776284 PMC2778845

[79] Stewart CR Stuart LM Wilkinson K van Gils JM Deng J. CD36 ligands promote sterile inflammation through assembly of a toll-like receptor 4 and 6 heterodimer. Nat Immunol. (2010) 11:155–61. 10.1038/ni.1836 20037584 PMC2809046

[80] Doi Y Mizuno T Maki Y Jin S Mizoguchi H. Microglia activated with the toll-like receptor 9 ligand CpG attenuate oligomeric amyloid {beta} neurotoxicity in *in vitro* and *in vivo* models of alzheimer's disease. Am J Pathol. (2009) 175:2121–32. 10.2353/ajpath.2009.090418 19834064 PMC2774075

[81] Tahara K Kim HD Jin JJ Maxwell JA Li L Fukuchi K. Role of toll-like receptor signalling in abeta uptake and clearance. Brain. (2006) 129:3006–19. 10.1093/brain/awl249 16984903 PMC2445613

[82] Iribarren P Chen K Hu J Gong W Cho EH. CpG-containing oligodeoxynucleotide promotes microglial cell uptake of amyloid beta 1-42 peptide by up-regulating the expression of the G-protein- coupled receptor mFPR2. FASEB J. (2005) 19:2032–4. 10.1096/fj.05-4578fje 16219804

[83] Chen K Iribarren P Hu J Chen J Gong W. Activation of toll-like receptor 2 on microglia promotes cell uptake of alzheimer disease-associated amyloid beta peptide. J Biol Chem. (2006) 281:3651–9. 10.1074/jbc.M508125200 16339765

[84] Chen K Huang J Liu Y Gong W Cui Y Wang JM. Synergy of TRIF-dependent TLR3 and MyD88-dependent TLR7 in up-regulating expression of mouse FPR2, a promiscuous G-protein-coupled receptor, in microglial cells. J Neuroimmunol. (2009) 213:69–77. 10.1016/j.jneuroim.2009.05.018 19559490 PMC2761824

[85] DiCarlo G Wilcock D Henderson D Gordon M Morgan D. Intrahippocampal LPS injections reduce abeta load in APP+PS1 transgenic mice. Neurobiol Aging. (2001) 22:1007–12. 10.1016/S0197-4580(01)00292-5 11755009

[86] Herber DL Roth LM Wilson D Wilson N Mason JE. Time-dependent reduction in abeta levels after intracranial LPS administration in APP transgenic mice. Exp Neurol. (2004) 190:245–53. 10.1016/j.expneurol.2004.07.007 15473997

[87] Malm TM Koistinaho M Parepalo M Vatanen T Ooka A. Bone-marrow-derived cells contribute to the recruitment of microglial cells in response to beta-amyloid deposition in APP/PS1 double transgenic alzheimer mice. Neurobiol Dis. (2005) 18:134–42. 10.1016/j.nbd.2004.09.009 15649704

[88] Michaud JP Halle M Lampron A Theriault P Prefontaine P. Toll-like receptor 4 stimulation with the detoxified ligand monophosphoryl lipid A improves alzheimer's disease-related pathology. Proc Natl Acad Sci USA. (2013) 110:1941–6. 10.1073/pnas.1215165110 23322736 PMC3562771

[89] Scholtzova H Kascsak RJ Bates KA Boutajangout A Kerr DJ. Induction of toll-like receptor 9 signaling as a method for ameliorating alzheimer's disease-related pathology. J Neurosci. (2009) 29:1846–54. 10.1523/JNEUROSCI.5715-08.2009 19211891 PMC2699573

[90] Scholtzova H Chianchiano P Pan J Sun Y Goni F. Amyloid beta and tau alzheimer's disease related pathology is reduced by toll-like receptor 9 stimulation. Acta Neuropathol Commun. (2014) 2:101. 10.1186/PREACCEPT-2151623761356337 25178404 PMC4171548

[91] Scholtzova H Do E Dhakal S Sun Y Liu S. Innate immunity stimulation via toll-like receptor 9 ameliorates vascular amyloid pathology in tg-SwDI mice with associated cognitive benefits. J Neurosci. (2017) 37:936–59. 10.1523/JNEUROSCI.1967-16.2016 28123027 PMC5296786

[92] Qiao X Cummins DJ Paul SM. Neuroinflammation-induced acceleration of amyloid deposition in the APPV717F transgenic mouse. Eur J Neurosci. (2001) 14:474–82. 10.1046/j.0953-816x.2001.01666.x 11553297

[93] Sheng JG Bora SH Xu G Borchelt DR Price DL Koliatsos VE. Lipopolysaccharide-induced-neuroinflammation increases intracellular accumulation of amyloid precursor protein and amyloid beta peptide in APPswe transgenic mice. Neurobiol Dis. (2003) 14:133–45. 10.1016/S0969-9961(03)00069-X 13678674

[94] McAlpine FE Lee JK Harms AS Ruhn KA Blurton-Jones M. Inhibition of soluble TNF signaling in a mouse model of alzheimer's disease prevents pre-plaque amyloid-associated neuropathology. Neurobiol Dis. (2009) 34:163–77. 10.1016/j.nbd.2009.01.006 19320056 PMC2948857

[95] Song M Jin J Lim JE Kou J Pattanayak A. TLR4 mutation reduces microglial activation, increases abeta deposits and exacerbates cognitive deficits in a mouse model of alzheimer's disease. J Neuroinflammation. (2011) 8:92. 10.1186/1742-2094-8-92 21827663 PMC3169468

[96] Jin JJ Kim HD Maxwell JA Li L Fukuchi K. Toll-like receptor 4-dependent upregulation of cytokines in a transgenic mouse model of alzheimer's disease. J Neuroinflammation. (2008) 5:23. 10.1186/1742-2094-5-23 18510752 PMC2430555

[97] Richard KL Filali M Prefontaine P Rivest S. Toll-like receptor 2 acts as a natural innate immune receptor to clear amyloid beta 1-42 and delay the cognitive decline in a mouse model of alzheimer's disease. J Neurosci. (2008) 28:5784–93. 10.1523/JNEUROSCI.1146-08.2008 18509040 PMC6670789

[98] Reed-Geaghan EG Reed QW Cramer PE Landreth GE. Deletion of CD14 attenuates alzheimer's disease pathology by influencing the brain's inflammatory milieu. J Neurosci. (2010) 30:15369–73. 10.1523/JNEUROSCI.2637-10.2010 21084593 PMC2997622

[99] Lim JE Song M Jin J Kou J Pattanayak A. The effects of MyD88 deficiency on exploratory activity, anxiety, motor coordination, and spatial learning in C57BL/6 and APPswe/PS1dE9 mice. Behav Brain Res. (2012) 227:36–42. 10.1016/j.bbr.2011.10.027 22051943 PMC3242934

[100] Hao W Liu Y Liu S Walter S Grimm MO. Myeloid differentiation factor 88-deficient bone marrow cells improve alzheimer's disease-related symptoms and pathology. Brain. (2011) 134:278–92. 10.1093/brain/awq325 21115468

[101] Hotamisligil GS. Inflammation and metabolic disorders. Nature. (2006) 444:860–7. 10.1038/nature05485 17167474

[102] Osborn O Olefsky JM. The cellular and signaling networks linking the immune system and metabolism in disease. Nat Med. (2012) 18:363–74. 10.1038/nm.2627 22395709

[103] Takeda S Sato N Uchio-Yamada K Sawada K Kunieda T. Diabetes-accelerated memory dysfunction via cerebrovascular inflammation and abeta deposition in an alzheimer mouse model with diabetes. Proc Natl Acad Sci USA. (2010) 107:7036–41. 10.1073/pnas.1000645107 20231468 PMC2872449

[104] Julien C Tremblay C Phivilay A Berthiaume L Emond V. High-fat diet aggravates amyloid-beta and tau pathologies in the 3xTg-AD mouse model. Neurobiol Aging. (2010) 31:1516–31. 10.1016/j.neurobiolaging.2008.08.022 18926603

[105] Libby P Ridker PM Hansson GK. Progress and challenges in translating the biology of atherosclerosis. Nature. (2011) 473:317–25. 10.1038/nature10146 21593864

[106] Hulsmans M Holvoet P. MicroRNA-containing microvesicles regulating inflammation in association with atherosclerotic disease. Cardiovasc Res. (2013) 100:7–18. 10.1093/cvr/cvt161 23774505

[107] Li L Cao D Garber DW Kim H Fukuchi K. Association of aortic atherosclerosis with cerebral beta-amyloidosis and learning deficits in a mouse model of alzheimer's disease. Am J Pathol. (2003) 163:2155–64. 10.1016/S0002-9440(10)63572-9 14633589 PMC1892402

[108] Tilvis RS Kahonen-Vare MH Jolkkonen J Valvanne J Pitkala KH Strandberg TE. Predictors of cognitive decline and mortality of aged people over a 10-year period. J Gerontol A Biol Sci Med Sci. (2004) 59:268–74. 10.1093/gerona/59.3.M268 15031312

[109] Kuo HK Yen CJ Chang CH Kuo CK Chen JH Sorond F. Relation of C-reactive protein to stroke, cognitive disorders, and depression in the general population: systematic review and meta-analysis. Lancet Neurol. (2005) 4:371–80. 10.1016/S1474-4422(05)70099-5 15907742

[110] Laurin D David Curb J Masaki KH White LR Launer LJ. Midlife C-reactive protein and risk of cognitive decline: a 31-year follow-up. Neurobiol Aging. (2009) 30:1724–7. 10.1016/j.neurobiolaging.2008.01.008 18316138 PMC7477790

[111] Tan ZS Beiser AS Vasan RS Roubenoff R Dinarello CA. Inflammatory markers and the risk of alzheimer disease: the framingham study. Neurology. (2007) 68:1902–8. 10.1212/01.wnl.0000263217.36439.da 17536046

[112] Singh-Manoux A Dugravot A Brunner E Kumari M Shipley M. Interleukin-6 and C-reactive protein as predictors of cognitive decline in late midlife. Neurology. (2014) 83:486–93. 10.1212/WNL.0000000000000665 24991031 PMC4141998

[113] Shen XN Niu LD Wang YJ Cao XP Liu Q. Inflammatory markers in alzheimer's disease and mild cognitive impairment: a meta-analysis and systematic review of 170 studies. J Neurol Neurosurg Psychiatry. (2019) 90:590–8. 10.1136/jnnp-2018-319148 30630955

[114] Balistreri CR Caruso C Listi F Colonna-Romano G Lio D Candore G. LPS-mediated production of pro/anti-inflammatory cytokines and eicosanoids in whole blood samples: biological effects of +896A/G TLR4 polymorphism in a sicilian population of healthy subjects. Mech Ageing Dev. (2011) 132:86–92. 10.1016/j.mad.2010.12.005 21238472

[115] Xu XM Ning YC Wang WJ Liu JQ Bai XY. Anti-inflamm-aging effects of long-term caloric restriction via overexpression of SIGIRR to inhibit NF-kappaB signaling pathway. Cell Physiol Biochem. (2015) 37:1257–70. 10.1159/000430248 26431348

[116] Michelsen KS Wong MH Shah PK Zhang W Yano J. Lack of toll-like receptor 4 or myeloid differentiation factor 88 reduces atherosclerosis and alters plaque phenotype in mice deficient in apolipoprotein E. Proc Natl Acad Sci USA. (2004) 101:10679–84. 10.1073/pnas.0403249101 15249654 PMC489994

[117] Kim JJ Sears DD. TLR4 and insulin resistance. Gastroenterol Res Pract. (2010) 2010:212563. 10.1155/2010/212563 20814545 PMC2931384

[118] Maheshwari P Eslick GD. Bacterial infection and alzheimer's disease: a meta-analysis. J Alzheimers Dis. (2015) 43:957–66. 10.3233/JAD-140621 25182736

[119] Bu XL Yao XQ Jiao SS Zeng F Liu YH. A study on the association between infectious burden and alzheimer's disease. Eur J Neurol. (2015) 22:1519–25. 10.1111/ene.12477 24910016

[120] McManus RM Higgins SC Mills KH Lynch MA. Respiratory infection promotes T cell infiltration and amyloid-beta deposition in APP/PS1 mice. Neurobiol Aging. (2014) 35:109–21. 10.1016/j.neurobiolaging.2013.07.025 23993702

[121] Lee JW Lee YK Yuk DY Choi DY Ban SB. Neuro-inflammation induced by lipopolysaccharide causes cognitive impairment through enhancement of beta-amyloid generation. J Neuroinflammation. (2008) 5:37. 10.1186/1742-2094-5-37 18759972 PMC2556656

[122] Kahn MS Kranjac D Alonzo CA Haase JH Cedillos RO. Prolonged elevation in hippocampal abeta and cognitive deficits following repeated endotoxin exposure in the mouse. Behav Brain Res. (2012) 229:176–84. 10.1016/j.bbr.2012.01.010 22249135

[123] Krstic D Madhusudan A Doehner J Vogel P Notter T. Systemic immune challenges trigger and drive alzheimer-like neuropathology in mice. J Neuroinflammation. (2012) 9:151. 10.1186/1742-2094-9-151 22747753 PMC3483167

[124] Kitazawa M Oddo S Yamasaki TR Green KN LaFerla FM. Lipopolysaccharide-induced inflammation exacerbates tau pathology by a cyclin-dependent kinase 5-mediated pathway in a transgenic model of alzheimer's disease. J Neurosci. (2005) 25:8843–53. 10.1523/JNEUROSCI.2868-05.2005 16192374 PMC6725603

[125] Xin YR Jiang JX Hu Y Pan JP Mi XN. The immune system drives synapse loss during lipopolysaccharide-induced learning and memory impairment in mice. Front Aging Neurosci. (2019) 11:279. 10.3389/fnagi.2019.00279 31803043 PMC6873885

[126] Liu J Wang D Li SQ Yu Y Ye RD. Suppression of LPS-induced tau hyperphosphorylation by serum amyloid A. J Neuroinflammation. (2016) 13:28. 10.1186/s12974-016-0493-y 26838764 PMC4736117

[127] Roe AD Staup MA Serrats J Sawchenko PE Rissman RA Lipopolysaccharide-induced tau phosphorylation and kinase activity–modulation, but not mediation, by corticotropin-releasing factor receptors. Eur J Neurosci. (2011) 34:448–56. 10.1111/j.1460-9568.2011.07764.x 21722209 PMC3148267

[128] Ishida N Ishihara Y Ishida K Tada H Funaki-Kato Y. Periodontitis induced by bacterial infection exacerbates features of alzheimer's disease in transgenic mice. NPJ Aging Mech Dis. (2017) 3:15. 10.1038/s41514-017-0015-x 29134111 PMC5673943

[129] Wu Z Ni J Liu Y Teeling JL Takayama F. Cathepsin B plays a critical role in inducing alzheimer's disease-like phenotypes following chronic systemic exposure to lipopolysaccharide from porphyromonas gingivalis in mice. Brain Behav Immun. (2017) 65:350–61. 10.1016/j.bbi.2017.06.002 28610747

[130] Zakaria R Wan Yaacob WM Othman Z Long I Ahmad AH Al-Rahbi B. Lipopolysaccharide-induced memory impairment in rats: a model of alzheimer's disease. Physiol Res. (2017) 66:553–65. 10.33549/physiolres.933480 28406691

[131] Schreuder L Eggen BJ Biber K Schoemaker RG Laman JD de Rooij SE. Pathophysiological and behavioral effects of systemic inflammation in aged and diseased rodents with relevance to delirium: a systematic review. Brain Behav Immun. (2017) 62:362–81. 10.1016/j.bbi.2017.01.010 28088641

[132] Neher JJ Cunningham C. Priming microglia for innate immune memory in the brain. Trends Immunol. (2019) 40:358–74. 10.1016/j.it.2019.02.001 30833177

[133] Batista CRA Gomes GF Candelario-Jalil E Fiebich BL de Oliveira ACP. Lipopolysaccharide-induced neuroinflammation as a bridge to understand neurodegeneration. Int J Mol Sci. (2019) 20:2293. 10.3390/ijms20092293 31075861 PMC6539529

[134] Chakravarty S Herkenham M. Toll-like receptor 4 on nonhematopoietic cells sustains CNS inflammation during endotoxemia, independent of systemic cytokines. J Neurosci. (2005) 25:1788–96. 10.1523/JNEUROSCI.4268-04.2005 15716415 PMC6725921

[135] Wendeln AC Degenhardt K Kaurani L Gertig M Ulas T. Innate immune memory in the brain shapes neurological disease hallmarks. Nature. (2018) 556:332–8. 10.1038/s41586-018-0023-4 29643512 PMC6038912

[136] Chovatiya R Medzhitov R. Stress, inflammation, and defense of homeostasis. Mol Cell. (2014) 54:281–8. 10.1016/j.molcel.2014.03.030 24766892 PMC4048989

[137] Guo H Callaway JB Ting JP. Inflammasomes: mechanism of action, role in disease, and therapeutics. Nat Med. (2015) 21:677–87. 10.1038/nm.3893 26121197 PMC4519035

[138] Swanson KV Deng M Ting JP. The NLRP3 inflammasome: molecular activation and regulation to therapeutics. Nat Rev Immunol. (2019) 19:477–89. 10.1038/s41577-019-0165-0 31036962 PMC7807242

[139] Guerreiro R Bras J. The age factor in alzheimer's disease. Genome Med. (2015) 7:106. 10.1186/s13073-015-0232-5 26482651 PMC4617238

[140] Baker DJ Petersen RC. Cellular senescence in brain aging and neurodegenerative diseases: evidence and perspectives. J Clin Invest. (2018) 128:1208–16. 10.1172/JCI95145 29457783 PMC5873891

[141] Youm YH Grant RW McCabe LR Albarado DC Nguyen KY. Canonical Nlrp3 inflammasome links systemic low-grade inflammation to functional decline in aging. Cell Metab. (2013) 18:519–32. 10.1016/j.cmet.2013.09.010 24093676 PMC4017327

[142] Sierra A Gottfried-Blackmore AC McEwen BS Bulloch K. Microglia derived from aging mice exhibit an altered inflammatory profile. Glia. (2007) 55:412–24. 10.1002/glia.20468 17203473

[143] Kritsilis MV Rizou S Koutsoudaki PN Evangelou K Gorgoulis VG. Ageing, cellular senescence and neurodegenerative disease. Int J Mol Sci. (2018) 19:2937. 10.3390/ijms19102937 30261683 PMC6213570

[144] Wu Z Sun L Hashioka S Yu S Schwab C. Differential pathways for interleukin-1beta production activated by chromogranin A and amyloid beta in microglia. Neurobiol Aging. (2013) 34:2715–25. 10.1016/j.neurobiolaging.2013.05.018 23831373

[145] Halle A Hornung V Petzold GC Stewart CR Monks BG. The NALP3 inflammasome is involved in the innate immune response to amyloid-beta. Nat Immunol. (2008) 9:857–65. 10.1038/ni.1636 18604209 PMC3101478

[146] Venegas C Kumar S Franklin BS Dierkes T Brinkschulte R. Microglia-derived ASC specks cross-seed amyloid-beta in alzheimer's disease. Nature. (2017) 552:355–61. 10.1038/nature25158 29293211

[147] Heneka MT Kummer MP Stutz A Delekate A Schwartz S. NLRP3 is activated in alzheimer's disease and contributes to pathology in APP/PS1 mice. Nature. (2013) 493:674–8. 10.1038/nature11729 23254930 PMC3812809

[148] Shaftel SS Griffin WS O'Banion MK. The role of interleukin-1 in neuroinflammation and alzheimer disease: an evolving perspective. J Neuroinflammation. (2008) 5:7. 10.1186/1742-2094-5-7 18302763 PMC2335091

[149] Lim JE Kou J Song M Pattanayak A Jin J. MyD88 deficiency ameliorates beta-amyloidosis in an animal model of alzheimer's disease. Am J Pathol. (2011) 179:1095–103. 10.1016/j.ajpath.2011.05.045 21763676 PMC3157279

[150] Ising C Venegas C Zhang S Scheiblich H Schmidt SV. NLRP3 inflammasome activation drives tau pathology. Nature. (2019) 575:669–73. 10.1038/s41586-019-1769-z 31748742 PMC7324015

[151] Heneka MT McManus RM Latz E. Inflammasome signalling in brain function and neurodegenerative disease. Nat Rev Neurosci. (2018) 19:610–21. 10.1038/s41583-018-0055-7 30206330

[152] Zhu W Cao FS Feng J Chen HW Wan JR. NLRP3 inflammasome activation contributes to long-term behavioral alterations in mice injected with lipopolysaccharide. Neuroscience. (2017) 343:77–84. 10.1016/j.neuroscience.2016.11.037 27923741 PMC5349320

[153] Voet S Mc Guire C Hagemeyer N Martens A Schroeder A. A20 critically controls microglia activation and inhibits inflammasome-dependent neuroinflammation. Nat Commun. (2018) 9:2036. 10.1038/s41467-018-04376-5 29789522 PMC5964249

[154] Dang R Guo YY Zhang K Jiang P Zhao MG. Predictable chronic mild stress promotes recovery from LPS-induced depression. Mol Brain. (2019) 12:42. 10.1186/s13041-019-0463-2 31053149 PMC6500057

[155] Gong W Zhang S Zong Y Halim M Ren Z. Involvement of the microglial NLRP3 inflammasome in the anti-inflammatory effect of the antidepressant clomipramine. J Affect Disord. (2019) 254:15–25. 10.1016/j.jad.2019.05.009 31082627

[156] Tejera D Mercan D Sanchez-Caro JM Hanan M Greenberg D. Systemic inflammation impairs microglial abeta clearance through NLRP3 inflammasome. EMBO J. (2019) 38:e101064. 10.15252/embj.2018101064 31359456 PMC6717897

[157] He Y Hara H Nunez G. Mechanism and regulation of NLRP3 inflammasome activation. Trends Biochem Sci. (2016) 41:1012–21. 10.1016/j.tibs.2016.09.002 27669650 PMC5123939

[158] Fernandes-Alnemri T Kang S Anderson C Sagara J Fitzgerald KA Alnemri ES. Cutting edge: TLR signaling licenses IRAK1 for rapid activation of the NLRP3 inflammasome. J Immunol. (2013) 191:3995–9. 10.4049/jimmunol.1301681 24043892 PMC3924784

[159] Bauernfeind FG Horvath G Stutz A Alnemri ES MacDonald K. Cutting edge: NF-kappaB activating pattern recognition and cytokine receptors license NLRP3 inflammasome activation by regulating NLRP3 expression. J Immunol. (2009) 183:787–91. 10.4049/jimmunol.0901363 19570822 PMC2824855

[160] Patel MN Carroll RG Galvan-Pena S Mills EL Olden R. Inflammasome priming in sterile inflammatory disease. Trends Mol Med. (2017) 23:165–80. 10.1016/j.molmed.2016.12.007 28109721

[161] Venegas C Heneka MT. Inflammasome-mediated innate immunity in alzheimer's disease. FASEB J. (2019) 33:13075–84. 10.1096/fj.201900439 31702392

[162] Parajuli B Sonobe Y Horiuchi H Takeuchi H Mizuno T Suzumura A. Oligomeric amyloid beta induces IL-1beta processing via production of ROS: implication in alzheimer's disease. Cell Death Dis. (2013) 4:e975. 10.1038/cddis.2013.503 24357806 PMC3877570

[163] Nakanishi A Kaneko N Takeda H Sawasaki T Morikawa S. Amyloid beta directly interacts with NLRP3 to initiate inflammasome activation: identification of an intrinsic NLRP3 ligand in a cell-free system. Inflamm Regen. (2018) 38:27. 10.1186/s41232-018-0085-6 30459926 PMC6231249

[164] Grebe A Hoss F Latz E. NLRP3 inflammasome and the IL-1 pathway in atherosclerosis. Circ Res. (2018) 122:1722–40. 10.1161/CIRCRESAHA.118.311362 29880500

[165] Kohl J. The role of complement in danger sensing and transmission. Immunol Res. (2006) 34:157–76. 10.1385/IR:34:2:157 16760575

[166] Holers VM. Complement and its receptors: new insights into human disease. Annu Rev Immunol. (2014) 32:433–59. 10.1146/annurev-immunol-032713-120154 24499275

[167] Laumonnier Y Karsten CM Kohl J. Novel insights into the expression pattern of anaphylatoxin receptors in mice and men. Mol Immunol. (2017) 89:44–58. 10.1016/j.molimm.2017.05.019 28600003

[168] Veerhuis R Nielsen HM Tenner AJ. Complement in the brain. Mol Immunol. (2011) 48:1592–603. 10.1016/j.molimm.2011.04.003 21546088 PMC3142281

[169] Stephan AH Barres BA Stevens B. The complement system: an unexpected role in synaptic pruning during development and disease. Annu Rev Neurosci. (2012) 35:369–89. 10.1146/annurev-neuro-061010-113810 22715882

[170] Stephan AH Madison DV Mateos JM Fraser DA Lovelett EA. A dramatic increase of C1q protein in the CNS during normal aging. J Neurosci. (2013) 33:13460–74. 10.1523/JNEUROSCI.1333-13.2013 23946404 PMC3742932

[171] Datta D Leslie SN Morozov YM Duque A Rakic P. Classical complement cascade initiating C1q protein within neurons in the aged rhesus macaque dorsolateral prefrontal cortex. J Neuroinflammation. (2020) 17:8. 10.1186/s12974-019-1683-1 31906973 PMC6945481

[172] Shi Q Colodner KJ Matousek SB Merry K Hong S. Complement C3-deficient mice fail to display age-related hippocampal decline. J Neurosci. (2015) 35:13029–42. 10.1523/JNEUROSCI.1698-15.2015 26400934 PMC6605437

[173] DeKosky ST Scheff SW. Synapse loss in frontal cortex biopsies in alzheimer's disease: correlation with cognitive severity. Ann Neurol. (1990) 27:457–64. 10.1002/ana.410270502 2360787

[174] Terry RD Masliah E Salmon DP Butters N DeTeresa R. Physical basis of cognitive alterations in alzheimer's disease: synapse loss is the major correlate of cognitive impairment. Ann Neurol. (1991) 30:572–80. 10.1002/ana.410300410 1789684

[175] Lambert JC Heath S Even G Campion D Sleegers K. Genome-wide association study identifies variants at CLU and CR1 associated with alzheimer's disease. Nat Genet. (2009) 41:1094–9. 10.1038/ng.439 19734903

[176] Ishii T Haga S. Immuno-electron-microscopic localization of complements in amyloid fibrils of senile plaques. Acta Neuropathol. (1984) 63:296–300. 10.1007/BF00687336 6382906

[177] Rogers J Cooper NR Webster S Schultz J McGeer PL. Complement activation by beta-amyloid in alzheimer disease. Proc Natl Acad Sci USA. (1992) 89:10016–20. 10.1073/pnas.89.21.10016 1438191 PMC50268

[178] Webster S Lue LF Brachova L Tenner AJ McGeer PL. Molecular and cellular characterization of the membrane attack complex, C5b-9, in alzheimer's disease. Neurobiol Aging. (1997) 18:415–21. 10.1016/S0197-4580(97)00042-0 9330973

[179] Stoltzner SE Grenfell TJ Mori C Wisniewski KE Wisniewski TM. Temporal accrual of complement proteins in amyloid plaques in down's syndrome with alzheimer's disease. Am J Pathol. (2000) 156:489–99. 10.1016/S0002-9440(10)64753-0 10666378 PMC1850044

[180] Litvinchuk A Wan YW Swartzlander DB Chen F Cole A. Complement C3aR inactivation attenuates tau pathology and reverses an immune network deregulated in tauopathy models and alzheimer's disease. Neuron. (2018) 100:1337–53.e5. 10.1016/j.neuron.2018.10.031 30415998 PMC6309202

[181] Yang LB Li R Meri S Rogers J Shen Y. Deficiency of complement defense protein CD59 may contribute to neurodegeneration in alzheimer's disease. J Neurosci. (2000) 20:7505–9. 10.1523/JNEUROSCI.20-20-07505.2000 11027207 PMC6772855

[182] Hakobyan S Harding K Aiyaz M Hye A Dobson R. Complement biomarkers as predictors of disease progression in alzheimer's disease. J Alzheimers Dis. (2016) 54:707–16. 10.3233/JAD-160420 27567854

[183] Hu WT Watts KD Tailor P Nguyen TP Howell JC. CSF complement 3 and factor H are staging biomarkers in alzheimer's disease. Acta Neuropathol Commun. (2016) 4:14. 10.1186/s40478-016-0277-8 26887322 PMC4758165

[184] Rasmussen KL Nordestgaard BG Frikke-Schmidt R Nielsen SF. An updated alzheimer hypothesis: complement C3 and risk of alzheimer's disease-A cohort study of 95,442 individuals. Alzheimers Dement. (2018) 14:1589–601. 10.1016/j.jalz.2018.07.223 30243924

[185] Winston CN Goetzl EJ Schwartz JB Elahi FM Rissman RA. Complement protein levels in plasma astrocyte-derived exosomes are abnormal in conversion from mild cognitive impairment to alzheimer's disease dementia. Alzheimers Dement. (2019) 11:61–6. 10.1016/j.dadm.2018.11.002 31032394 PMC6477776

[186] Morgan AR Touchard S Leckey C O'Hagan C Nevado-Holgado AJ. Inflammatory biomarkers in alzheimer's disease plasma. Alzheimers Dement. (2019) 15:776–87. 10.1016/j.jalz.2019.03.007 31047856 PMC6565806

[187] Fonseca MI Zhou J Botto M Tenner AJ. Absence of C1q leads to less neuropathology in transgenic mouse models of alzheimer's disease. J Neurosci. (2004) 24:6457–65. 10.1523/JNEUROSCI.0901-04.2004 15269255 PMC6729885

[188] Hong S Beja-Glasser VF Nfonoyim BM Frouin A Li S. Complement and microglia mediate early synapse loss in alzheimer mouse models. Science. (2016) 352:712–6. 10.1126/science.aad8373 27033548 PMC5094372

[189] Dejanovic B Huntley MA De Maziere A Meilandt WJ Wu T. Changes in the synaptic proteome in tauopathy and rescue of tau-induced synapse loss by C1q antibodies. Neuron. (2018) 100:1322–36.e7. 10.1016/j.neuron.2018.10.014 30392797

[190] Haga S Ikeda K Sato M Ishii T. Synthetic alzheimer amyloid beta/A4 peptides enhance production of complement C3 component by cultured microglial cells. Brain Res. (1993) 601:88–94. 10.1016/0006-8993(93)91698-R 8431789

[191] Shi Q Chowdhury S Ma R Le KX Hong S. Complement C3 deficiency protects against neurodegeneration in aged plaque-rich APP/PS1 mice. Sci Transl Med. (2017) 9:eaaf6295. 10.1126/scitranslmed.aaf6295 28566429 PMC6936623

[192] Wu T Dejanovic B Gandham VD Gogineni A Edmonds R. Complement C3 is activated in human AD brain and is required for neurodegeneration in mouse models of amyloidosis and tauopathy. Cell Rep. (2019) 28:2111–23.e6. 10.1016/j.celrep.2019.07.060 31433986

[193] Allendorf DH Puigdellivol M Brown GC. Activated microglia desialylate their surface, stimulating complement receptor 3-mediated phagocytosis of neurons. Glia. (2019) 68:989–98. 10.1002/glia.23757 31774586 PMC7079032

[194] Fonseca MI Ager RR Chu SH Yazan O Sanderson SD. Treatment with a C5aR antagonist decreases pathology and enhances behavioral performance in murine models of alzheimer's disease. J Immunol. (2009) 183:1375–83. 10.4049/jimmunol.0901005 19561098 PMC4067320

[195] Benoit ME Hernandez MX Dinh ML Benavente F Vasquez O Tenner AJ. C1q-induced LRP1B and GPR6 proteins expressed early in alzheimer disease mouse models, are essential for the C1q-mediated protection against amyloid-beta neurotoxicity. J Biol Chem. (2013) 288:654–65. 10.1074/jbc.M112.400168 23150673 PMC3537064

[196] Maier M Peng Y Jiang L Seabrook TJ Carroll MC Lemere CA. Complement C3 deficiency leads to accelerated amyloid beta plaque deposition and neurodegeneration and modulation of the microglia/macrophage phenotype in amyloid precursor protein transgenic mice. J Neurosci. (2008) 28:6333–41. 10.1523/JNEUROSCI.0829-08.2008 18562603 PMC3329761

[197] Wyss-Coray T Yan F Lin AH Lambris JD Alexander JJ. Prominent neurodegeneration and increased plaque formation in complement-inhibited alzheimer's mice. Proc Natl Acad Sci USA. (2002) 99:10837–42. 10.1073/pnas.162350199 12119423 PMC125059

[198] Bodea LG Wang Y Linnartz-Gerlach B Kopatz J Sinkkonen L. Neurodegeneration by activation of the microglial complement-phagosome pathway. J Neurosci. (2014) 34:8546–56. 10.1523/JNEUROSCI.5002-13.2014 24948809 PMC6608212

[199] Liddelow SA Guttenplan KA Clarke LE Bennett FC Bohlen CJ. Neurotoxic reactive astrocytes are induced by activated microglia. Nature. (2017) 541:481–7. 10.1038/nature21029 28099414 PMC5404890

[200] Zhang X Kimura Y Fang C Zhou L Sfyroera G. Regulation of toll-like receptor-mediated inflammatory response by complement *in vivo*. Blood. (2007) 110:228–36. 10.1182/blood-2006-12-063636 17363730 PMC1896115

[201] Pope MR Hoffman SM Tomlinson S Fleming SD. Complement regulates TLR4-mediated inflammatory responses during intestinal ischemia reperfusion. Mol Immunol. (2010) 48:356–64. 10.1016/j.molimm.2010.07.004 20800895 PMC2993765

[202] O'Barr S Cooper NR. The C5a complement activation peptide increases IL-1beta and IL-6 release from amyloid-beta primed human monocytes: implications for alzheimer's disease. J Neuroimmunol. (2000) 109:87–94. 10.1016/S0165-5728(00)00291-5 10996210

[203] Triantafilou K Hughes TR Triantafilou M Morgan BP. The complement membrane attack complex triggers intracellular Ca^2+^ fluxes leading to NLRP3 inflammasome activation. J Cell Sci. (2013) 126:2903–13. 10.1242/jcs.124388 23613465

[204] Seow V Lim J Iyer A Suen JY Ariffin JK. Inflammatory responses induced by lipopolysaccharide are amplified in primary human monocytes but suppressed in macrophages by complement protein C5a. J Immunol. (2013) 191:4308–16. 10.4049/jimmunol.1301355 24043889

[205] Ricklin D Reis ES Lambris JD. Complement in disease: a defence system turning offensive. Nat Rev Nephrol. (2016) 12:383–401. 10.1038/nrneph.2016.70 27211870 PMC4974115

[206] Lesiak AJ Neumaier JF. RiboTag: not lost in translation. Neuropsychopharmacology. (2016) 41:374–6. 10.1038/npp.2015.262 26657954 PMC4677145

[207] Haimon Z Volaski A Orthgiess J Boura-Halfon S Varol D. Re-evaluating microglia expression profiles using RiboTag and cell isolation strategies. Nat Immunol. (2018) 19:636–44. 10.1038/s41590-018-0110-6 29777220 PMC5986066

[208] Penney J Ralvenius WT Tsai LH. Modeling alzheimer's disease with iPSC-derived brain cells. Mol Psychiatry. (2020) 25:148–67. 10.1038/s41380-019-0468-3 31391546 PMC6906186

